# The impact of pre-transplantation nephrectomy on quality of life in patients with autosomal dominant polycystic kidney disease

**DOI:** 10.1007/s00345-023-04349-4

**Published:** 2023-03-17

**Authors:** Paul Geertsema, Ron T. Gansevoort, Lisanne P. J. Brenkman, Shosha E. I. Dekker, Damia V. P. Eleveld, Johan W. de Fijter, Anna M. Leliveld, Maya Levy, Esther Meijer, Robert A. Pol, Emmelien E. M. Schillern, Jan-Stephan F. Sanders, Niek F. Casteleijn

**Affiliations:** 1 Department of Nephrology, University Medical Center Groningen, University of Groningen, Groningen, The Netherlands; 2 Department of Nephrology, Leiden University Medical Center, Leiden, The Netherlands; 3 Department of Urology, Expertise Center for Polycystic Diseases, University Medical Center Groningen, University of Groningen, PO Box 30.001, 9700 RB Groningen, The Netherlands; 4 Department of Surgery, University Medical Center Groningen, University of Groningen, Groningen, The Netherlands

**Keywords:** ADPKD, Nephrectomy, Quality of life, Kidney transplantation, Polycystic kidney disease

## Abstract

**Purpose:**

In selected ADPKD patients, a nephrectomy is required in the work-up for a kidney transplantation. Because the impact of this procedure is unknown, we investigated the effect of pre-transplantation nephrectomy on quality of life in this group.

**Methods:**

In this retrospective cohort study all ADPKD patients, ≥ 18 years, who received a kidney transplantation in 2 ADPKD expertise centers between January 2000 and January 2016, were asked to participate. Quality of life was assessed using three validated questionnaires on three time points. Nephrectomy was performed in preparation for transplantation.

**Results:**

Two hundred seventy-six ADPKD patients (53 ± 9 years, 56.2% male) were included. 98 patients (35.5%) underwent native nephrectomy in preparation for transplantation, of which 43 underwent bilateral nephrectomy. Pre-transplantation, ADPKD-IS scores were worse in the nephrectomy group vs. no-nephrectomy group (physical: 2.9 vs. 2.3, *p* < 0.001; emotional: 2.0 vs. 1.8, *p* = 0.03; fatigue: 3.0 vs. 2.3, *p* = 0.01). Post-transplantation and post-nephrectomy, ADPKD-IS scores improved significantly in both groups, with a significantly higher improvement in the nephrectomy group. During follow-up, all scores were still better compared to pre-transplantation. Observed physical QoL (ADPKD-IS physical 1.3 vs. 1.7, *p* = 0.04; SF-36 physical 50.0 vs. 41.3, *p* = 0.03) was better post-transplantation after bilateral nephrectomy compared to unilateral nephrectomy. In retrospect, 19.7% of patients would have liked to undergo a nephrectomy, while the decision not to perform nephrectomy was made by the treating physician.

**Conclusion:**

This study shows that pre-transplantation nephrectomy improves quality of life in selected ADPKD patients. Bilateral nephrectomy may be preferred, although the risk of additional complications should be weighted.

**Supplementary Information:**

The online version contains supplementary material available at 10.1007/s00345-023-04349-4.

## Introduction

Autosomal dominant polycystic kidney disease (ADPKD) is characterized by the formation of numerous renal cysts, resulting in progressive kidney growth and kidney function decline [1]. Although the rate of disease progression is variable in ADPKD patients, the majority of patients ultimately need kidney replacement therapy [2]. In such patients, kidney transplantation is the first-choice treatment modality [3, 4]. During the work-up for a kidney transplantation, a (bilateral) nephrectomy is indicated in a substantial part of ADPKD patients because of lack of space for the kidney allograft [5–9]. Other indications for pre-emptive nephrectomy may be recurrent (cyst) infections, persistent cyst hemorrhage or refractory pain. Nephrectomy is regarded to be a safe procedure in ADPKD patients, and can be performed either by a (robot assisted) laparoscopic or by an open approach [6, 7]. Recent studies showed that complications did not differ by timing of nephrectomy and renal graft survival is not affected by this procedure [7, 10].

It is known that patients with severe chronic kidney disease experience a high symptom burden, for instance fatigue as well as impaired physical and mental condition [11, 12]. This results in a reduced quality of life, which is further decreased when patients become dialysis dependent [11, 13]. After kidney transplantation, quality of life significantly increases in most of these patients due to an improvement of their physical condition [14]. Similar to non-ADPKD patients with chronic kidney disease, ADPKD patients may also experience a decrease in quality of life when disease progresses, which can even be more affected by volume-related complaints due to their increase in total kidney and liver volume [15].

To our knowledge, it has not been studied before whether pre-transplantation nephrectomy has a positive effect on quality of life. In this study we aimed to investigate the short and long-term impact of pre-transplantation nephrectomy on quality of life in transplanted ADPKD patients. Our secondary aims were to investigate the difference of the impact on quality of life of a unilateral and bilateral procedure and to investigate the patient experience.

## Materials and methods

### Study design and study population

In this retrospective cohort study, all ADPKD patients, ≥ 18 years, who received a kidney transplantation in the University Medical Centers of Groningen and Leiden between January 2000 and January 2016, were asked to participate. Exclusion criteria for the present analysis were a follow-up period ≤ 12 months after kidney transplantation and a previous kidney transplantation. In addition, patients were excluded when a nephrectomy was performed either post-transplantation, or more than one year pre-transplantation with an indication not related to a future transplantation. The study protocol was reviewed by the institutional review board of the University Medical Center Groningen and deemed exempt of approval because of its non-interventional and non-burdensome character (METc 2018/422).

### Data collection

All eligible patients received an invitation letter and printed questionnaire by mail with a return envelope. The invitation letter and questionnaire were reviewed by six specialists involved in the care of ADPKD patients and seven members of the Dutch Kidney Patient Association (among which ADPKD patients) for content, length, comprehension, language and answer options. Patients who did not respond within 3 weeks were sent a written reminder. Collected data consisted of patient characteristics, nephrectomy, transplantation, quality of life and patient experience. Patients in the work-up for kidney transplantation are seen by a multidisciplinary team that includes a transplant surgeon. In case this specialist judged, based on the supposed availability of enough space for the transplant kidney in the iliac fossa, that a nephrectomy was needed, a referral to a urologist followed, who performed the actual nephrectomy. Patients could have one or multiple indications for nephrectomy, such as lack of space for kidney allograft, recurrent cyst infections, refractory pain, or persistent cyst hemorrhage. Quality of life was assessed using one symptom score and three validated questionnaires: the ADPKD-Impact scale (IS), Patient Health Questionnaire-9 (PHQ-9) and Short Form-36 (SF-36) [16–18]. The ADPKD-IS is a validated assessment of ADPKD-related health aspects, consisting of 14 items representing 3 domains: physical, emotional, and fatigue [16]. This score ranges from 1 to 5, with a higher score indicating lower ADPKD related health [16]. The PHQ-9 is a screening tool for depression, consisting of 9 items representing 9 symptoms of depression, scored from 0 to 27, with a higher score reflecting diminished mental health [17]. Lastly, the SF-36 assesses general well-being, and its scores were aggregated into a physical component and a mental component score [18]. There summary scores were scored from 0 to 100, with a higher score reflecting better quality of life [18]. To record the severity of gastrointestinal (GI) complaints, a symptom survey was included containing questions about 9 symptoms: abdominal fullness, poor tolerance to food, early satiety, anorexia, involuntary weight loss, nausea, vomiting, gastric complaints and obstipation. The severity of each symptom was assessed using a 5-point Likert scale (not at all, a little, somewhat, a lot, very much). A GI symptom score was calculated from these 9 GI items by summing the scores of all symptoms and converting this to a 0 to 100 scale, reflecting no symptoms to maximal discomfort. All patients were asked to fill out these questionnaires retrospectively with respect to three different time points: 12 months before transplantation, 12 months after transplantation and at follow-up (i.e., the moment of filling in the questionnaires). To measure patient experience and satisfaction with the delivered care, a questionnaire was added which consisted of 3 items for patients who did not undergo nephrectomy and 14 items for patients who did (Supplementary File 1). All study data were collected and managed using REDCap (Research Electronic Data Capture) [19].

### Statistical analysis

Normally distributed continuous data and non-normally distributed continuous data were expressed as mean ± standard deviation (SD) or median [inter quartile range], respectively. Nominal data are presented as percentage (%). Differences in patient characteristics and outcome parameters between groups were analyzed using Student’s t-test for normally distributed continuous data, Mann–Whitney *U* test for non-normally distributed continuous data, and chi-square test for categorical data. A two-sided *p* < 0.05 was considered to indicate statistical significance. Patients were categorized into two groups: no-nephrectomy and nephrectomy in preparation for transplantation, which will be referred to as the nephrectomy group. Changes in outcome parameters between time points within groups were analyzed using Wilcoxon’s sign rank test for non-normally distributed continuous data, and McNemar’s test for categorical data. Secondary analyses were performed in patients subdivided according to whether they had a unilateral or a bilateral nephrectomy in preparation for transplantation, and according to whether patients received a nephrectomy and transplantation in the University Medical Center Groningen or in the Leiden University Medical Center. All statistical analyses were performed using SPSS (IBM SPSS Statistics for Windows, Version 23.0. Armonk, NY: IBM Corp).

## Results

**Fig. 1:**
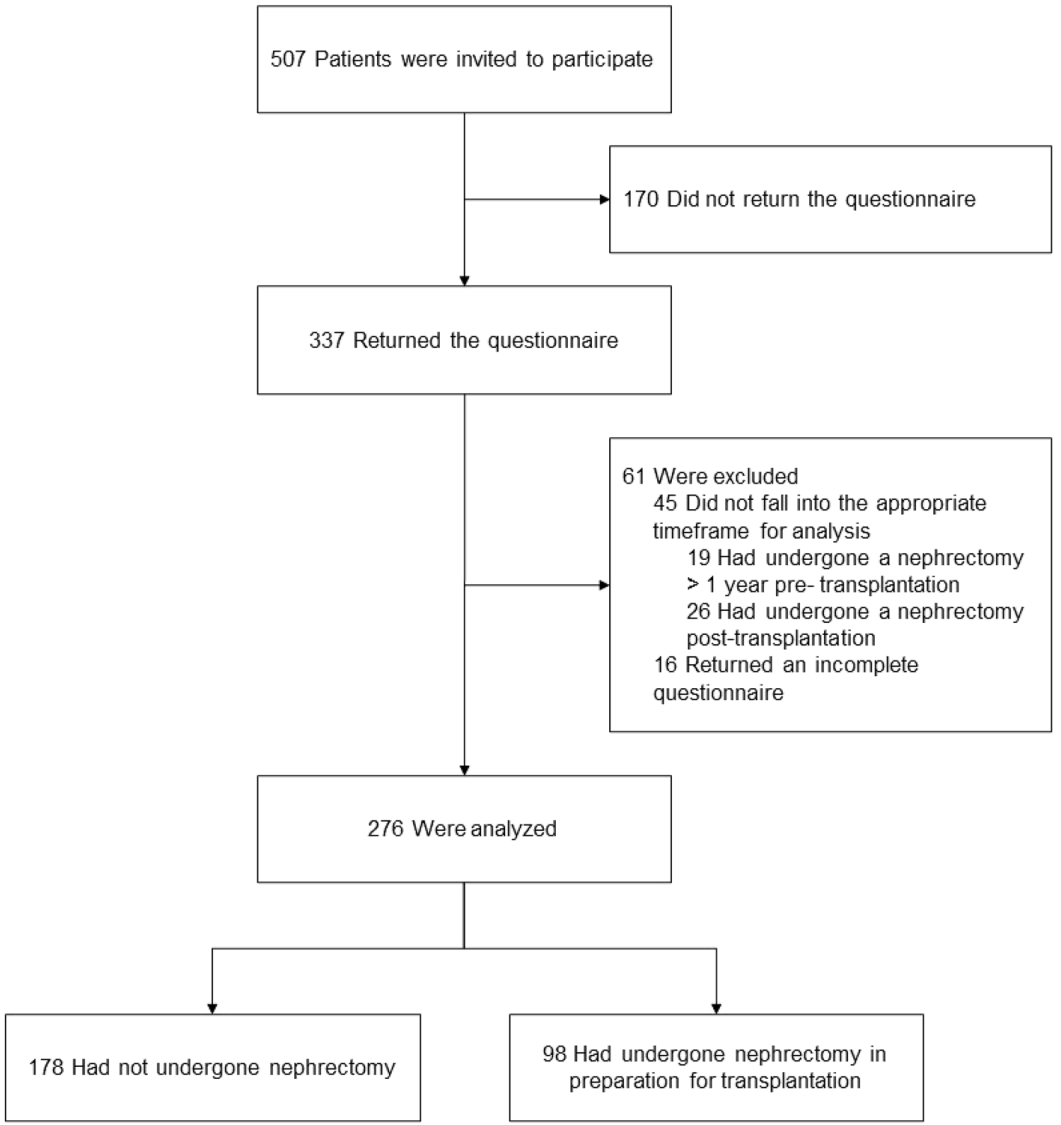
Patient inclusion flowchart

In total, 507 patients were invited to participate, of whom 337 returned the questionnaire, indicating a 66.5% response rate. No data are known on the non-responders. After exclusion of 16 patients who returned an incomplete questionnaire and 45 patients who met exclusion criteria, 276 transplanted ADPKD patients were included in the present study (Fig. 1). Of these patients, 98 patients underwent nephrectomy in preparation for transplantation. Overall age at transplantation was 53 ± 9 years, 56.2% were male and mean BMI was 26.2 ± 4.4 (Table 1). No differences in patient characteristics were found between the nephrectomy and no-nephrectomy group, except for need for pre-transplantation dialysis (58.8% vs. 91.8%, *p* =  < 0.001).

**Table 1:** Patient characteristics

	No nephrectomy (*n* = 178)	Nephrectomy in preparation for transplantation (*n* = 98)	*P* val.
Age at transplantation (yrs)	54 ± 10	53 ± 7	0.9
Male sex, *n (%)*	96 (53.9)	59 (60.2)	0.2
Height (cm)	175 ± 10	177 ± 10	0.1
Weight (kg)	79 ± 13	84 ± 19	0.1
BMI (kg/m^2^)	25.8 ± 3.8	27.0 ± 5.2	0.1
Comorbidities, *n (%)*			
Diabetes	23 (12.9)	19 (19.4)	0.4
Cardiovascular disease	26 (14.6)	22 (22.4)	0.3
COPD	3 (1.7)	4 (4.1)	0.4
Previous malignancy	8 (4.5)	8 (8.2)	0.4
Dialysis before transplantation, *n (%)*	104 (58.8)	90 (91.8)	< 0.001
Dialysis modality, *n (%)*			
Peritoneal dialysis	45 (25.3)	26 (26.5)	0.9
Hemodialysis	58 (32.6)	78 (79.6)	< 0.001
Exercise, *n (%)*	93 (55.4)	57 (60.9)	0.3
Frequency (times per week)	2.5 ± 1.9	2.5 ± 1.6	0.5
Hours per week	3.6 ± 3.7	3.1 ± 2.5	0.6

*BMI* Body Mass Index, *COPD* Chronic obstructive pulmonary disease

Of the patients in the nephrectomy group, 43 (43.8%) underwent a bilateral procedure. Following a first unilateral procedure, 14 patients underwent subsequently a contralateral nephrectomy. The most common indication for nephrectomy was lack of space (*n* = 68), followed by recurrent cyst infections (*n* = 18) and refractory pain (*n* = 9).

### Quality of life before transplantation

Before transplantation, ADPKD-IS physical, emotional and fatigue scores in the overall study population were 2.4 [1.7–3.1], 1.8 [1.3–2.3] and 2.7 [2.0–3.7], respectively (Table 2). Overall physical and mental component scores were 37.2 and 50.8, respectively, and the PHQ-9 and GI-score were 4.0 and 10.0. ADPKD-IS scores were lower before transplantation in the group that first had a nephrectomy compared to those who did not undergo a nephrectomy (physical: 2.9 vs. 2.3, *p* < 0.001; emotional: 2.0 vs. 1.8, *p* = 0.03; fatigue: 3.0 vs. 2.3, *p* = 0.01), as well as the physical component score, mental component score, PHQ-9 and GI-score (33.6 vs. 38.4, *p* = 0.002; 48.7 vs. 51.7, *p* = 0.03; 5.0 vs. 4.0, *p* = 0.01; 15.6 vs. 8.9, *p* = 0.001) (Suppl. Table 1).

**Table 2:** Quality of life assessments before and after transplantation and after long-term follow-up

	Before transplantation	After transplantation	Follow-up	*P* val.^1^	*P *val.^2^	*P* val.^3^
Overall (*n* = 276)						
ADPKD-IS physical	2.4 [1.7–3.1]	1.4 [1.1–2.1]	1.6 [1.1–2.3]	< 0.001	< 0.001	0.045
ADPKD-IS emotional	1.8 [1.3–2.3]	1.3 [1.0–1.5]	1.3 [1.0–1.7]	< 0.001	< 0.001	0.2
ADPKD-IS fatigue	2.7 [2.0–3.7]	1.3 [1.0–2.0]	1.7 [1.0–2.3]	< 0.001	< 0.001	0.02
PHQ-9 score	4.0 [2.0–8.0]	1.0 [0–4.0]	2.0 [0–5.0]	< 0.001	< 0.001	0.001
SF-36 Physical component score	37.2 [30.5–45.5]	51.7 [42.8–55.8]	48.7 [35.6–55.1]	< 0.001	< 0.001	< 0.001
SF-36 Mental component score	50.8 [42.0–56.8]	56.6 [50.8–60.3]	56.6 [50.7–59.9]	< 0.001	< 0.001	0.7
Gastrointestinal symptom score	10.0 [2.2–24.4]	2.2 [0–6.7]	2.2 [0–8.9]	< 0.001	< 0.001	0.4
No nephrectomy (*n* = 178)						
ADPKD-IS physical	2.3 [1.6–2.9]	1.4 [1.1–2.1]	1.4 [1.1–2.3]	< 0.001	< 0.001	0.3
ADPKD-IS emotional	1.8 [1.3–2.3]	1.3 [1.0–1.5]	1.3 [1.0–1.5]	< 0.001	< 0.001	0.8
ADPKD-IS fatigue	2.3 [1.7–3.3]	1.7 [1.0–2.0]	1.7 [1.0–2.3]	< 0.001	< 0.001	0.2
PHQ-9 score	4.0 [2.0–7.0]	1.0 [0–4.0]	2.0 [0–5.0]	< 0.001	< 0.001	0.02
SF-36 Physical component score	38.4 [32.2–47.4]	52.1 [43.8–56.1]	49.7 [36.9–55.4]	< 0.001	< 0.001	0.001
SF-36 Mental component score	51.7 [43.7–56.8]	56.2 [50.0–59.7]	55.1 [50.2–59.9]	< 0.001	< 0.001	0.7
Gastrointestinal symptom score	8.9 [2.2–20.0]	2.2 [0–6.7]	2.2 [0–8.3]	< 0.001	< 0.001	0.7
Nephrectomy in preparation for transplantation (*n* = 98)						
ADPKD-IS physical	2.9 [2.0–3.5]	1.4 [1.1–2.1]	1.6 [1.0–2.6]	< 0.001	< 0.001	0.04
ADPKD-IS emotional	2.0 [1.5–2.5]	1.3 [1.0–1.8]	1.3 [1.0–1.8]	< 0.001	< 0.001	0.1
ADPKD-IS fatigue	3.0 [2.0–4.0]	1.3 [1.0–2.0]	1.7 [1.0–2.7]	< 0.001	< 0.001	0.02
PHQ-9 score	5.0 [3.0–11.0]	1.0 [0–4.0]	2.0 [0–6.0]	< 0.001	< 0.001	0.02
SF-36 Physical component score	33.6 [27.4–42.7]	50.9 [41.2–55.1]	45.8 [33.0–53.7]	< 0.001	< 0.001	0.001
SF-36 Mental component score	48.7 [37.0–54.6]	58.3 [51.8–60.6]	57.3 [53.0–60.1]	< 0.001	< 0.001	0.9
Gastrointestinal symptom score	15.6 [4.4–31.1]	2.2 [0–6.7]	3.3 [0–8.9]	< 0.001	< 0.001	0.4

*IS* Impact Scale, *PHQ* Patient Health Questionnaire, *SF-36* Short Form 36; ^1^ comparison between before transplantation and after transplantation; ^2^ comparison between before transplantation and follow-up; and ^3^, comparison between after transplantation and follow-up. A lower score in the ADPKD-IS, PHQ-9 and gastrointestinal scale indicates a better quality of life. A lower score in de SF-36 scores indicates a worse quality of life

### Quality of life after transplantation and at long-term follow-up

**Fig. 2:**
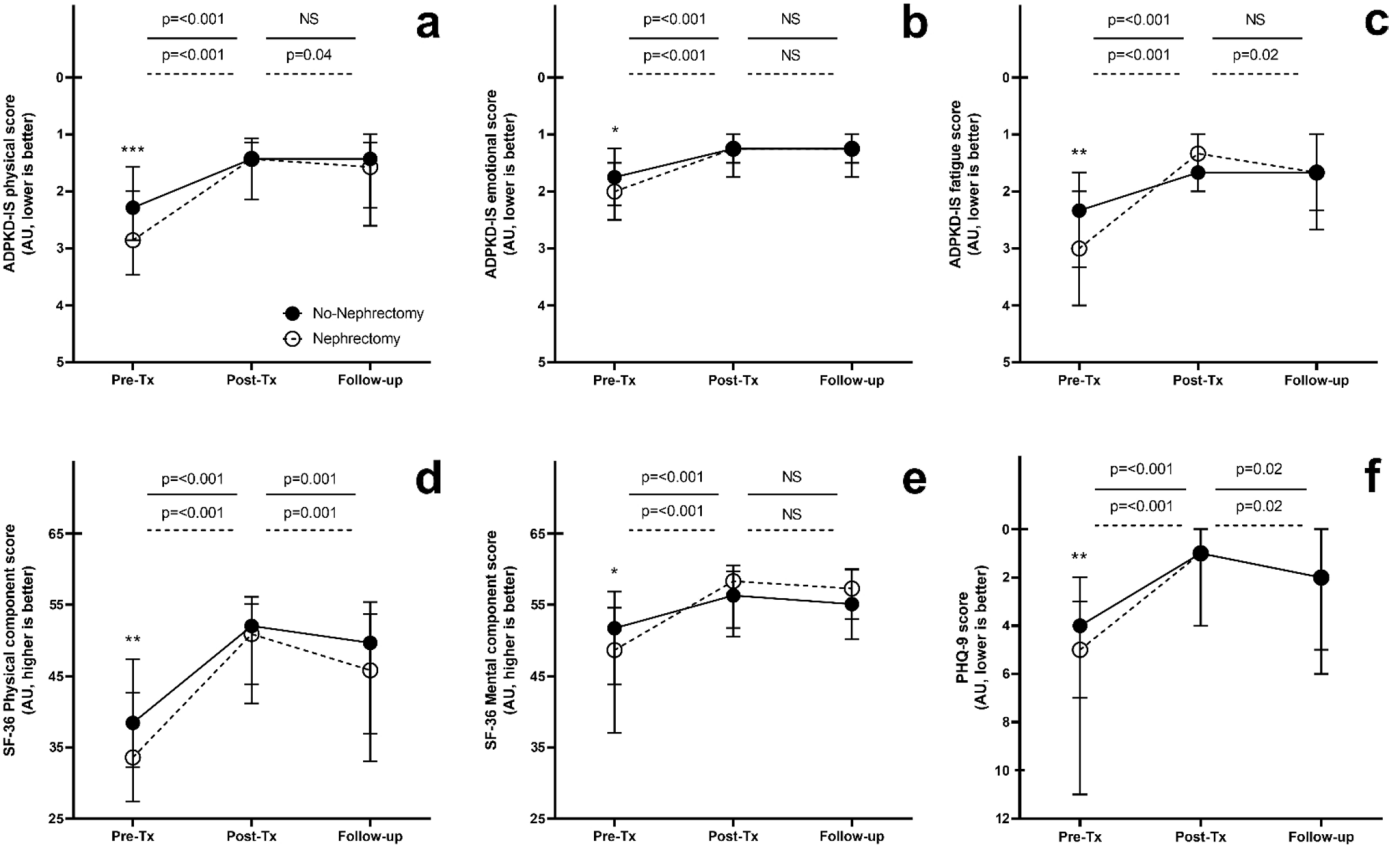
Quality of life assessment before and after transplantation, depicted in the no nephrectomy (*n* = 178) and nephrectomy group (*n* = 98) separately. **a** ADPKD-IS physical; **b** ADPKD-IS emotional; **c** ADPKD-IS fatigue; **d** SF-36 Physical component score; **e** SF-36 Mental component score; **f** PHQ-9. *Abbreviations are: IS *Impact scale, *SF-36* Short Form 36, *AU* artificial unit, *Pre-Tx* 12 months before transplantation, *Post-Tx* 12 months after transplantation. Comparison between both groups on several time points: *, *p* =  < 0.05, **, *p* =  < 0.01 ***, *p* =  < 0.001. A higher level on the Y-axis depicts a higher quality of life

After pre-transplantation nephrectomy and transplantation, there were no longer any differences in the ADPKD-IS scores between the nephrectomy group and the no-nephrectomy group (1.4 vs. 1.4, *p* = 0.9; 1.3 vs. 1.3, *p* = 0.9; and 1.7 vs. 1.3, *p* = 0.9, respectively) (Fig. 2). Similar findings were observed for the other quality of live assessments. At long-term follow-up (7.7 ± 4.6 yrs), quality of life was still comparable between both groups.

### Quality of life between time points

Quality of life, as observed by the ADPKD-IS physical, ADPKD-IS emotional and ADPKD-IS fatigue scores significantly improved after transplantation in both the no-nephrectomy group (change − 0.9, *p* < 0.001; − 0.5, *p* < 0.001; and − 0.6, *p* < 0.001, respectively) and the nephrectomy group (− 1.5, *p* < 0.001; − 0.7, *p* < 0.001; and − 1.7, *p* < 0.001, respectively) (Table 2.). However, quality of life increased significantly more in the nephrectomy group compared to the no-nephrectomy group, resulting in a comparable quality of life after transplantation and nephrectomy, as well as at long-term follow-up. Also the PHQ-9 score, physical component score, mental component score, and GI-symptom score significantly improved after transplantation in both groups (− 3.0, + 13.7, + 4.5, − 6.7 in the no-nephrectomy group; − 4.0, + 17.3, + 9.6, − 13.4 in the nephrectomy group), again with a greater improvement in the nephrectomy group compared to the no-nephrectomy group (Fig. 2). At long-term follow-up the PHQ-9 and physical component scores were slightly decreased compared to one year after transplantation in the no-nephrectomy group (*p* = 0.02, *p* = 0.001, respectively) as well as the ADPKD-IS physical, ADPKD-IS fatigue, PHQ-9, and physical component score in the nephrectomy group (*p* = 0.04, *p* = 0.02, *p* = 0.02, *p* = 0.001, respectively). Of note, all scores were still significantly improved at long-term follow-up compared to pre-transplantation.

### Quality of life and unilateral versus bilateral nephrectomy

**Fig. 3:**
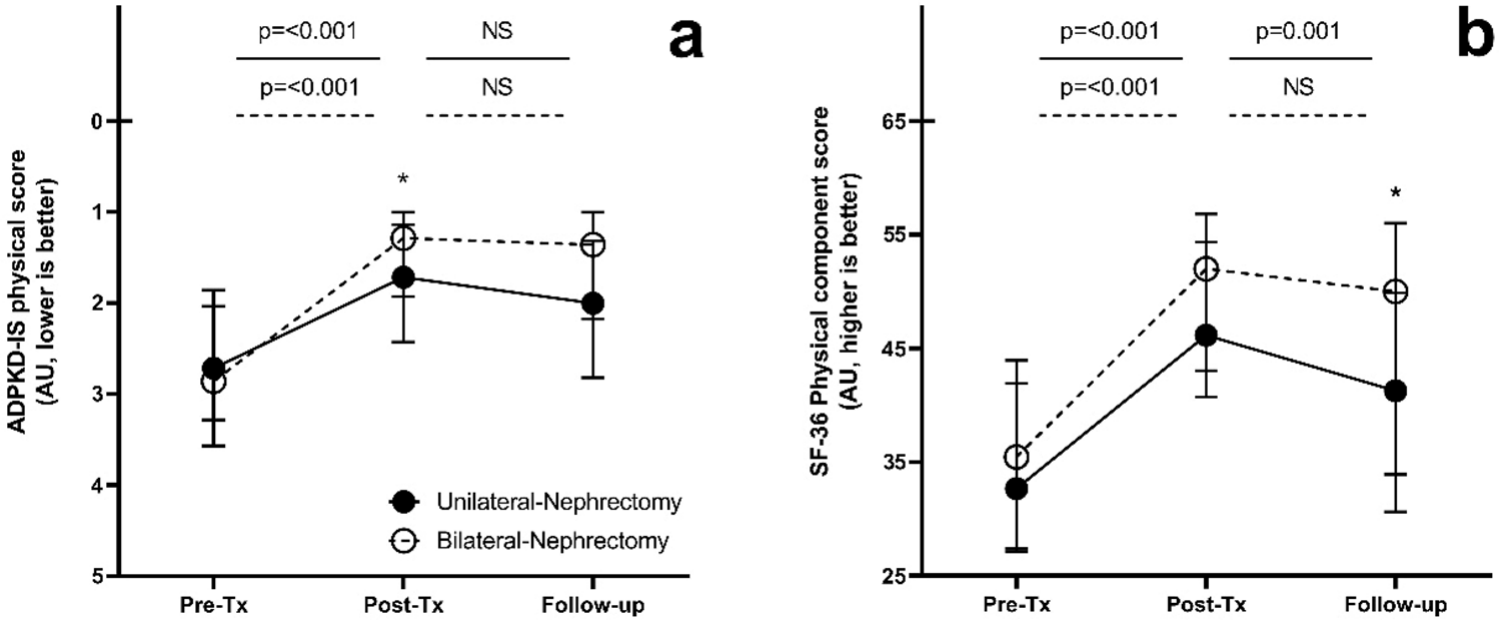
Quality of life assessment for unilateral (*n* = 54) and bilateral (*n* = 43) nephrectomy separately. **A** ADPKD-IS physical; **B** SF-36 Physical component score. *Abbreviations are: IS* Impact scale, *SF-36* Short Form 36, *AU* artificial unit, *Pre-Tx* 12 months before transplantation, *Post-Tx* 12 months after transplantation. Comparison between both groups on several time points: *, *p* =  < 0.05, **, *p* =  < 0.01 ***, *p* =  < 0.001. A higher level on the Y-axis depicts a higher quality of life

Patients who underwent a unilateral nephrectomy in preparation for transplantation had a similar quality of life before transplantation and nephrectomy compared to patients who underwent a bilateral nephrectomy (Table 3). After transplantation and at long-term follow-up, the physical quality of life scores of patients who had undergone a bilateral nephrectomy were marginally, but statistically significantly better compared to those who had undergone a unilateral nephrectomy. This is shown by the ADPKD-IS physical score and physical component score (1.3 vs. 1.7, *p* = 0.04; 50.0 vs. 41.3, *p* = 0.03, respectively) (Fig. 3). All other quality of life scores did not differ between groups, nor timepoints. When comparing quality of life of patients who received a transplantation and nephrectomy in the University Medical Center Groningen or Leiden University Medical Center, no center differences were found, except for the mental component score after transplantation, which differed between both centers (59.4 vs. 55.4, *p* = 0.04; Suppl. Table 2).

**Table 3:** Quality of life assessment in patients with unilateral and bilateral nephrectomy procedure

	Unilateral procedure (*n* = 54)*	Bilateral procedure (*n* = 43)*	*P* val
Before transplantation			
ADPKD-IS physical	2.7 [2.0–3.6]	2.9 [1.9–3.3]	0.8
ADPKD-IS emotional	2.0 [1.5–2.5]	2.0 [1.3–2.5]	0.9
ADPKD-IS fatigue	3.0 [2.0–3.75]	2.8 [2.0–4.0]	0.9
PHQ-9 score	5.0 [3.0–10.5]	5.0 [2.8–11.0]	0.9
SF-36 Physical component score	32.7 [27.1–41.9]	35.5 [27.4–43.9]	0.4
SF-36 Mental component score	48.7 [37.8–55.7]	48.7 [36.0–53.6]	0.7
Gastrointestinal symptom score	15.6 [8.9–29.4]	16.7 [4.4–32.2]	0.9
After transplantation			
ADPKD-IS physical	1.7 [1.1–2.4]	1.3 [1.0–1.9]	0.04
ADPKD-IS emotional	1.3 [1.0–1.8]	1.0 [1.0–1.6]	0.4
ADPKD-IS fatigue	1.5 [1.0–2.1]	1.3 [1.0–2.0]	0.4
PHQ-9 score	1.0 [0–4.3]	1.0 [0–4.0]	0.9
SF-36 Physical component score	46.2 [40.7–54.4]	52.0 [43.0–56.8]	0.1
SF-36 Mental component score	59.2 [52.4–61.5]	57.2 [51.2–60.0]	0.2
Gastrointestinal symptom score	2.2 [0–8.9]	2.2 [0–4.4]	0.2
Follow-up			
ADPKD-IS physical	2.0 [1.3–2.8]	1.4 [1.0–2.2]	0.1
ADPKD-IS emotional	1.3 [1.0–1.8]	1.3 [1.0–1.9]	0.7
ADPKD-IS fatigue	2.0 [1.3–2.7]	1.7 [1.0–2.5]	0.4
PHQ-9 score	2.5 [0–6.3]	2.0 [0.3–5.8]	0.8
SF-36 Physical component score	41.3 [30.6–49.9]	50.0 [33.9–56.0]	0.03
SF-36 Mental component score	58.2 [55.0–61.1]	56.1 [49.5–59.2]	0.1
Gastrointestinal symptom score	3.3 [0–8.9]	2.2 [0–5.6]	0.2

IS, Impact Scale; PHQ, Patient Health Questionnaire; SF-36, Short Form 36. A lower score in the ADPKD-IS, PHQ-9 and gastrointestinal scale indicates a better quality of life. A lower score in de SF-36 scores indicates a worse quality of life

^*^ The lateralization of one procedure was unknown

### Patient experience

During the study, patients were asked to review the peri-operative period of their transplantation. In the no-nephrectomy group, 31 (19.7%) patients indicated that, in retrospect, they would have rather liked to have one or both kidneys removed. The majority of this group (96.5%) stated that the decision not to remove a kidney was made by their treating physician (Table 4). Of note, only 17 (11.6%) patients indicated that they were not satisfied with the decision not to remove a kidney. In the group that did receive a nephrectomy, nearly all patients (*n* = 87, 94.6%) indicated that in retrospect, they would choose to undergo the procedure again (Suppl. Table 3). Seventy-five (83.3%) patients were satisfied with the long-term results of the nephrectomy, while only 6 (6.6%) were disappointed. Most patients (*n* = 64, 71.9%) indicated that the procedure met their expectations, although a substantial part of the patients (*n* = 44. 46.3%) experienced more pain than expected, albeit that this was only shortly after nephrectomy.

**Table 4:** Patient experience regarding the peri-operative transplantation time period in patients without nephrectomy (*n* = 178)

In retrospect, would you prefer that one or both kidneys was removed? *n(%)*	
▪ Yes	31 (19.7)
▪ No	126 (80.3)
The decision to remove a kidney was primarily the advice of the treating decision or your preferred own choice? *n(%)*	
▪ Own choice	6 (3.8)
▪ Advice treating physician	152 (96.5)
Are you still satisfied with the abovementioned decision? *n(%)*	
▪ Yes	130 (88.4)
▪ No	17 (11.6)

## Discussion

This study indicates that ADPKD patients who underwent a nephrectomy in preparation for transplantation had a lower quality of life compared to patients without nephrectomy. After nephrectomy and transplantation, quality of life significantly improved in these patients, resulting in a similar quality of life level in both groups at short as well as long-term follow-up. A bilateral procedure was associated with better physical quality of life after transplantation and nephrectomy, compared to a unilateral procedure. In retrospect, a relatively large percentage of patients who did not undergo a nephrectomy in the pre-transplantation work-up would have preferred to have one or both kidneys removed.

It is known that around 20–45% of the patients undergo nephrectomy before transplantation, mostly due to lack of space for the renal allograft [5–8, 20]. In our study, nephrectomy was performed in 44.5% of the patients, which is in line with current literature. As expected, the majority of nephrectomies were performed because of lack of space for the kidney allograft (69%). This number may be somewhat higher compared to other studies, that report that pre-transplantation nephrectomy is needed in 25–37% of the patients because of lack of space [7, 20]. We suggest that this could be explained by the fact that we pursue a restrictive approach in our centers, in which nephrectomy is performed only in patients with severe complaints, i.e., in case of serious volume related complaints, recurrent cyst infections, persistent cyst bleedings or chronic refractory pain [10]. Therefore, almost all nephrectomies were performed due to lack of space for the renal allograft, resulting in a relatively higher percentage.

Several studies assessed quality of life in the general population and found that the SF-36 physical component score is estimated around 49–50 and the mental component score around 52–54 [21, 22]. A previous study which used the SF-36 to measure quality of life in ADPKD patients with a eGFR between 20 and 44 ml/min/1.73m^2^ found lower physical component scores ranging between 48 and 51 and a lower mental component score ranging between 51 and 52 [23]. When kidney function declines further, physical quality of life also declines, as found by Erikson et al., who used the SF-12 to assess quality of life in ADPKD patients and found a physical component score of 43 and 35 in CKD 4–5 and dialysis patients, respectively [24]. In our study ADPKD patients experienced especially impaired physical quality of life pre-transplantation (physical component score 37) compared to the general population and ADPKD patients with an eGFR between 20 and 44 ml/min/1.73m2. However, quality of life was comparable to values from literature in ADPKD patients with CKD 4–5 and those on dialysis, indicating that we had a representative sample of ADPKD patients.

Several studies showed that kidney transplantation resulted in an improvement of quality of life. In a study of 1023 patients on dialysis, Jofre et al. found that patients who received a kidney transplant experienced a significant improvement of quality of life, especially male patients [25]. A cross-sectional study in 243 ADPKD patients found similar results, as they observed a difference of approximately 22% in the physical component score between dialysis and transplantation patients [24]. Using longitudinal data, we found an even greater difference of 28% and we can, therefore, confirm that transplantation improves quality of life, as we also see this improvement when only assessing the no-nephrectomy group.

It should be noted that a statistically significant difference in quality of life does not directly mean a clinically significant difference in quality of life. A tool to assess whether a difference in a quality of life score also indicates a clinical difference is called the minimal clinically important difference (MCID) [26]. For the SF-36 component scores the MCID has been determined to be 3 to 5 [26]. In our study, a difference in the physical component score of 4.8 was observed 1 year before transplantation between the no-nephrectomy and nephrectomy groups, indicating a clinically significant difference, whereas there was no difference in quality of life after transplantation. Therefore, it may be assumed that pre-transplantation nephrectomy has an additional positive effect on quality of life. A possible explanation for this improvement may be that nephrectomy reduced the high intra-abdominal volume, which has been associated with more pain, GI-symptoms and worse quality of life in other studies [15, 23]. By removing one or both polycystic kidneys, intra-abdominal volume will be reduced and quality of life may improve. Our data supports this assumption, because not only the physical health scores improved after nephrectomy and transplantation, also GI-symptoms as well as PHQ-9 scores increased.

A topic of debate is whether a unilateral or bilateral nephrectomy is the procedure of choice. Our study shows an association between a bilateral nephrectomy procedure and better physical quality of life, compared to a unilateral procedure. The difference in the physical component score between groups was 8.7 at long-term follow-up, which is considerably more than the MCID of 3–5, indicating that this difference is likely clinically relevant [26]. These data suggest that when a pre-transplantation nephrectomy is performed, a bilateral procedure may be the preferred choice. However, this decision should be weighed against the known risks of such a procedure. A number of studies noticed that nephrectomy in ADPKD had no negative impact on survival [7, 10, 27–29]. In a previous paper from our center we reported the perioperative aspects, as well as patient and graft survival data in ADPKD patients who underwent nephrectomy somewhere in the transplant process [10]. In the majority of patients, no peri-operative complications were observed (64.5%). In case there were complications, these were nearly always minor. In addition, no difference in patient and graft survival was noticed between patients with and without nephrectomy. Our results are in line with the findings in a large cohort study in 470 transplanted ADPKD patients in the United States [7]. Therefore, we consider nephrectomy in the pre-transplantation period as a low to medium risk procedure. Notwithstanding, there will always be some risk associated with nephrectomy of a polycystic kidney and therefore this procedure should only be performed on indication. As for a bilateral procedure, Kirkman et. al. found a 15% higher complication rate and a 15% higher mortality when compared to a unilateral nephrectomy [30]. Furthermore, bilateral pre-transplantation nephrectomy results in the patient becoming anephric, with the need for dialysis with a restricted fluid intake, which is more relevant side effect in ADPKD, as these patients in general have better preservation of residual renal function and diuresis [31]. Lastly, blood pressure regulation may be more challenging in anephric patients during dialysis [32].

For these reasons, we suggest that, when indicated, a stepwise approach may be appropriate, and to first perform a unilateral nephrectomy to make sufficient space for the kidney allograft and only when needed to perform a contralateral nephrectomy after transplantation. Several studies showed that a post-transplantation nephrectomy did not lead to a higher complication rate and did not compromise graft and patient survival [7, 10]. Another strategy would be the option of simultaneous bilateral nephrectomy and transplantation. It was shown in multiple studies that although simultaneous nephrectomy and transplantation results in a greater need for red blood cell transfusion and more complications in some studies, graft survival and mortality are similar in these patients [33, 34]. We think that such a complicated procedure might be feasible when a living donor is available, and the procedure can be planned. However, a substantial portion of patients receives a kidney of a deceased donor. These allografts are in general unplanned, creating logistical problems as two teams need to be present at the time of a simultaneous procedure: a transplant team and a nephrectomy team. For most hospitals it is impossible to have two teams available at all times. Furthermore, it is not known whether a post-transplantation or simultaneous transplantation and nephrectomy affects quality of life differently. This issue needs further investigation.

When we inquired about patient experience, we found that although patients had more pain shortly after the procedure than they expected, the majority of patients mentioned that they did not regret that they underwent a pre-transplantation nephrectomy. Of the patients in the no-nephrectomy group, almost 20% noticed that, in retrospect, they would have wanted to undergo a nephrectomy. A possible explanation for this may be that treating physicians do not inform patients of the possibility of a nephrectomy, as indicated by 97% of patients in the no-nephrectomy group when asked who took the decision not to perform a nephrectomy. Informing patients of the possibility of a nephrectomy, while highlighting the known positive and negative aspects, may result in a lower percentage of patients who would have preferred a nephrectomy. Given these data, we suggest that assessing patient preferences should be part of the shared decision-making process in the pre-transplantation period, wherein quality of life questionnaires should be used as diagnostic tools to assess whether patients may benefit from nephrectomy.

Our study has limitations. Due to its retrospective design, there may be a risk for recall bias. Second, our data may not be representative for the overall group of patients. However, of the 507 invited patients, 337 patients responded to our questionnaire. A response rate of 66% is in generally regarded to be satisfactory for questionnaires [35]. In addition, in the present study no data was available regarding peri-operative complications and graft failure. However, in another study of our institution with partly the same study population, we showed that the incidence of peri-operative complications was low and there was no difference in death censored graft loss and mortality between nephrectomy and no-nephrectomy groups [10]. The main strengths of our study are that we are the first to systematically study the effect of a pre-transplantation nephrectomy on quality of life, the use of validated questionnaires, the long-term follow-up and the additional assessment of the patient perspective.

In conclusion, this study shows that ADPKD patients who were selected to undergo a pre-transplantation nephrectomy experienced more complaints before transplantation compared to patients who did not undergo a nephrectomy, resulting in a lower quality of life. After nephrectomy and transplantation, quality of life improved significantly in these patients, resulting in a similar quality of life level in transplanted ADPKD patients who did or did not receive a nephrectomy at short as well as long-term follow-up. Bilateral nephrectomy may have additional benefit on physical quality of life compared to unilateral nephrectomy, although potential complications of such a more extensive procedure should be taken into account. A substantial percentage of patients would, in retrospect, have liked to undergo a nephrectomy, while the decision not to remove a kidney was made by the treating physician in almost all patients. We suggest to add assessment of quality of life and the patient perspective in the pre-transplantation work-up to consider objectively which ADPKD patients may benefit from pre-transplantation nephrectomy.

## Supplementary Information

Below is the link to the electronic supplementary material.

Supplementary file1 (DOCX 24 KB)

Supplementary file2 (PDF 563 KB)

## Data Availability

The data underlying this article will be shared upon reasonable request to the corresponding author.
