# Supplementary material for: The impact of pre-transplantation nephrectomy on quality of life in patients with autosomal dominant polycystic kidney disease
Source: World J Urol. 2023 Mar 17;41(4):1193–203. doi: 10.1007/s00345-023-04349-4 (PMC10160200; doi:10.1007/s00345-023-04349-4)
Supplement: Supplementary file 1 — Supplementary file1 (DOCX 24 KB) [file 345_2023_4349_MOESM1_ESM.docx]

| **Supplementary Table 1.** Quality of life between groups | | | |
| --- | --- | --- | --- |
|  | **No nephrectomy**  **(n = 178)** | **Nephrectomy in preparation for transplantation (n = 98)** | **P-val.** |
| **Before transplantation** |  |  |  |
| ADPKD-IS physical | 2.3 [1.6-2.9] | 2.9 [2.0-3.5] | <0.001 |
| ADPKD-IS emotional | 1.8 [1.3-2.3] | 2.0 [1.5-2.5] | 0.03 |
| ADPKD-IS fatigue | 2.3 [1.7-3.3] | 3.0 [2.0-4.0] | 0.01 |
| PHQ-9 score | 4.0 [2.0-7.0] | 5.0 [3.0-11.0] | 0.01 |
| SF-36 Physical component score | 38.4 [32.2-47.4] | 33.6 [27.4-42.7] | 0.002 |
| SF-36 Mental component score | 51.7 [43.7-56.8] | 48.7 [37.0-54.6] | 0.03 |
| Gastrointestinal symptom score | 8.9 [2.2-20.0] | 15.6 [4.4-31.1] | 0.001 |
| **After transplantation** |  |  |  |
| ADPKD-IS physical | 1.4 [1.1-2.1] | 1.4 [1.1-2.1] | 0.9 |
| ADPKD-IS emotional | 1.3 [1.0-1.5] | 1.3 [1.0-1.8] | 0.9 |
| ADPKD-IS fatigue | 1.7 [1.0-2.0] | 1.3 [1.0-2.0] | 0.9 |
| PHQ-9 score | 1.0 [0-4.0] | 1.0 [0-4.0] | 0.5 |
| SF-36 Physical component score | 52.1 [43.8-56.1] | 50.9 [41.2-55.1] | 0.1 |
| SF-36 Mental component score | 56.2 [50.0-59.7] | 58.3 [51.8-60.6] | 0.1 |
| Gastrointestinal symptom score | 2.2 [0-6.7] | 2.2 [0-6.7] | 0.6 |
| **Follow-up** |  |  |  |
| ADPKD-IS physical | 1.4 [1.1-2.3] | 1.6 [1.0-2.6] | 0.3 |
| ADPKD-IS emotional | 1.3 [1.0-1.5] | 1.3 [1.0-1.8] | 0.5 |
| ADPKD-IS fatigue | 1.7 [1.0-2.3] | 1.7 [1.0-2.7] | 0.4 |
| PHQ-9 score | 2.0 [0-5.0] | 2.0 [0-6.0] | 0.2 |
| SF-36 Physical component score | 49.7 [36.9-55.4] | 45.8 [33.0-53.7] | 0.1 |
| SF-36 Mental component score | 55.1 [50.2-59.9] | 57.3 [53.0-60.1] | 0.2 |
| Gastrointestinal symptom score | 2.2 [0-8.3] | 3.3 [0-8.9] | 0.9 |
| *Abbreviations are: IS, Impact scale; SF-36, Short Form 36. A lower score in the ADPKD-IS, PHQ-9 and gastrointestinal scale indicates a better quality of life. A lower score in de SF-36 scores indicates a worse quality of life. *, The lateralization of two procedures was unknown* | | | |

| **Supplemental Table 2.** Quality of life between nephrectomies performed in the University Medical Center Groningen and Leiden | | | |
| --- | --- | --- | --- |
|  | **Groningen (n = 34)** | **Leiden (n = 64)** | **P-val.** |
| **Before transplantation** |  |  |  |
| ADPKD-IS physical | 2.4 [1.7-3.1] | 2.4 [1.8-3.0] | 0.8 |
| ADPKD-IS emotional | 2.0 [1.3-2.3] | 1.8 [1.3-2.5] | 0.6 |
| ADPKD-IS fatigue | 2.7 [2.0-3.7] | 2.7 [2.0-3.8] | 0.9 |
| PHQ-9 score | 6.0 [3.0-9.0] | 5.0 [3.0-11.5] | 0.8 |
| SF-36 Physical component score | 32.6 [27.6-39.3] | 35.5 [27.3-45.2] | 0.3 |
| SF-36 Mental component score | 50.3 [37.1-57.3] | 48.6 [36.5-53.5] | 0.4 |
| Gastrointestinal symptom score | 15.6 [8.9-32.2] | 16.7 [4.4-30.6] | 0.9 |
| **After transplantation** |  |  |  |
| ADPKD-IS physical | 1.6 [1.1-2.3] | 1.4 [1.1-2.0] | 0.4 |
| ADPKD-IS emotional | 1.3 [1.0-1.5] | 1.3 [1.0-1.8] | 0.8 |
| ADPKD-IS fatigue | 1.7 [1.0-2.3] | 1.3 [1.0-2.0] | 0.8 |
| PHQ-9 score | 1.0 [0-4.0] | 1.0 [0-4.0 | 0.7 |
| SF-36 Physical component score | 43.6 [39.9-54.4] | 51.2 [44.6-55.8] | 0.3 |
| SF-36 Mental component score | 59.4 [54.4-62.2] | 55.4 [50.9-60.1] | 0.04 |
| Gastrointestinal symptom score | 3.3 [0-8.3] | 2.2 [0-5.6] | 0.5 |
| **Follow-up** |  |  |  |
| ADPKD-IS physical | 1.7 [1.1-2.6] | 1.4 [1.0-2.1] | 0.7 |
| ADPKD-IS emotional | 1.3 [1.0-1.5] | 1.3 [1.0-1.8] | 0.2 |
| ADPKD-IS fatigue | 1.7 [1.0-2.3] | 1.7 [1.0-2.3] | 0.7 |
| PHQ-9 score | 2.0 [0-6.0] | 2.0 [1.0-6.8] | 0.6 |
| SF-36 Physical component score | 41.2 [29.4-50.9] | 47.6 [33.9-54.6] | 0.1 |
| SF-36 Mental component score | 58.8 [52.1-61.3] | 56.9-53.6-59.1] | 0.2 |
| Gastrointestinal symptom score | 2.2 [0-8.9] | 4.4 [0-8.9] | 0.7 |
| *Abbreviations are: IS, Impact Scale; PHQ, Patient Health Questionnaire; SF-36,* *Short Form 36. A lower score in the ADPKD-IS, PHQ-9 and gastrointestinal scale indicates a better quality of life. A lower score in de SF-36 scores indicates a worse quality of life.* | | | |

| **Supplementary Table 3.** Patient experience in patients with nephrectomy | | |
| --- | --- | --- |
|  | Nephrectomy in preparation for transplantation  (n=98) | |
| Are you satisfied with the nephrectomy outcome after one year? *n(%)* |  | |
| - Dissatisfied | 9 (9.6) | |
| - No opinion | 9 (9.6) | |
| - Satisfied | 76 (80.9) | |
| Are you satisfied with the nephrectomy outcome after long-term follow up? *n(%)* |  | |
| - Dissatisfied | 6 (6.6) | |
| - No opinion | 9 (10.0) | |
| - Satisfied | 75 (83.3) | |
| Did the procedure meet your expectations? *n(%)* |  | |
| - Not at all/a little | 10 (11.2) | |
| - Somewhat | 15 (16.9) | |
| - A lot | 64 (71.9) | |
| In retrospect, would you have preferred to undergo a nephrectomy at an earlier time point? *n(%)* |  | |
| - No | 33 (35.1) | |
| - No opinion | 31 (33.0) | |
| - Yes | 30 (31.9) | |
| In retrospect, would you have preferred to undergo a nephrectomy at a later stage? *n(%)* |  | |
| - No | 56 (61.5) | |
| - No opinion | 27 (29.7) | |
| - Yes | 8 (8.8) | |
| Would you have preferred to have your kidney removed at the same time as the transplantation? *n(%)* |  | |
| - No | 39 (42.9) | |
| - No opinion | 31 (34.1) | |
| - Yes | 21 (23.1) | |
| Would you have preferred to have both kidneys removed at the same time? *n(%)* |  | |
| - No | 22 (26.8) | |
| - No opinion | 24 (29.3) | |
| - Yes | 36 (43.9) | |
| Did the pain experience meet your expectations after nephrectomy? *n(%)* |  | |
| - Yes | 36 (37.9) | |
| - No opinion | 15 (15.8) | |
| - No | 44 (46.3) | |
| Did your mental status improve after nephrectomy? *n(%)* |  | |
| - No | 14 (14.9) | |
| - No opinion | 27 (28.7) | |
| - Yes | 53 (56.4) | |
| When did you achieve a pain-free status after nephrectomy? *n(%)* |  | |
| - Within one week | 17 (18.1) | |
| - Within 1-4 weeks | 46 (46.9) | |
| - Within 4-8 weeks | 17 (18.1) | |
| - Within 8-16 weeks | 8 (8.5) | |
| - After more than 16 weeks | 6 (6.4) | |
| After how many months did your health status significantly improve after nephrectomy? | 3.0 [1.5-4.0] | |
| When did you completely recover after nephrectomy? *n(%)* |  |  |
| - Within one week | 0 (0.0) | |
| - Within 1-4 weeks | 17 (18.9) | |
| - Within 4-8 weeks | 25 (27.8) | |
| - Within 8-16 weeks | 29 (32.2) | |
| - After more than 16 weeks | 19 (21.1) | |
| In retrospect, would you choose to undergo a nephrectomy again? *n(%)* |  | |
| - No | 5 (5.4) | |
| - Yes | 87 (94.6) | |
| If not, why? *n(%)* |  | |
| - The outcome of the procedure | 1 (16.7) | |
| - Disappointing improvement of complaints | 1 (16.7) | |
| - Disappointing cosmetic results | 1 (16.7) | |
| - Disappointing improvement of pain complaints | 3 (50.0) | |
|  | | |
