# Supplementary material for: The impact of pre-transplantation nephrectomy on quality of life in patients with autosomal dominant polycystic kidney disease
Source: World J Urol. 2023 Mar 17;41(4):1193–203. doi: 10.1007/s00345-023-04349-4 (PMC10160200; doi:10.1007/s00345-023-04349-4)
Supplement: Supplementary file 2 — Supplementary file2 (PDF 563 KB) [file 345_2023_4349_MOESM2_ESM.pdf]

Geachte meneer/mevrouw,

Vanuit het expertisecentrum polycysteuze nierziekten van het Universitair Medisch Centrum Groningen willen wij u graag uitnodigen om bij te dragen aan een onderzoek naar patiënten met cystenieren (ook wel ADPKD genoemd). Onder begeleiding van en prof. Gansevoort (UMCG) en prof. de Fijter (LUMC) doen wij, voor de studie Geneeskunde, onderzoek naar de uitvoering en timing van het operatief verwijderen van een cystenier bij ADPKD patiënten. Voor zover wij begrepen hebben, is bij u een nier verwijderd en/of getransplanteerd. Voor ons onderzoek zijn we benieuwd wat voor invloed deze operatie(s) heeft/hebben gehad op uw leven.

Achtergrond
Veel patiënten met cystenieren zullen op een gegeven moment over moeten gaan op dialyse of een niertransplantatie ondergaan. In voorbereiding op een niertransplantatie worden bij sommige patiënten met cystenieren één of beide cystenieren operatief verwijderd. Zo'n operatie kan ook tijdens of na de niertransplantatie worden uitgevoerd. Enkele redenen om een cystenier te verwijderen zijn: klachten over onbehandelbare pijn, terugkerende ontstekingen of ruimtetekort voor de donornier. Wanneer hier geen sprake van is wordt een nierverwijdering vaak afgeraden, omdat deze operatie ook nodige risico's met zich meebrengt. Ziekenhuizen en artsen in Nederland zijn het echter niet altijd eens over wanneer en bij welke klachten een nierverwijdering plaats moet vinden; hierover bestaat momenteel geen landelijke richtlijn. Dit is onwenselijk; de zorg hierin de zorg wordt beter als patiënten in elk ziekenhuis zoveel mogelijk dezelfde, wetenschappelijk bewezen, behandeling krijgen. Er is alleen nog weinig bekend over dit onderwerp.

Wat wij doen
Het doel van het onderzoek is het schrijven van een landelijk protocol waarin staat bij welke patiënten één of beide cystenieren moeten worden verwijderd, en wanneer de ingreep het beste uitgevoerd kan worden. Daarbij letten we er onder andere op hoe patiënten zich voelen vóór en na de transplantatie of het verwijderen van de nier. Hiervoor hebben wij na toestemming van de Medisch Ethische Commissie samen met patiënten en artsen een vragenlijst gemaakt voor patiënten met ADPKD.

Hoe kunt u helpen?
U zou ons onderzoek enorm helpen door de bijgevoegde vragenlijst in te vullen en naar ons terug te zenden middels de bijgevoegde retourenvelop. Hiervoor is dus geen postzegel nodig. Het invullen van de vragenlijst kost ongeveer **een half uur**. Als het kan, adviseren we u om de vragenlijst samen met uw partner, een ander familielid of vriend(in) in te vullen. Er is maar een beperkte tijd voor ons onderzoek. Het zou ons daarom ontzettend helpen als u de vragenlijst zo snel mogelijk naar ons terug stuurt.

Alvast veel dank voor uw bijdrage!

Met vriendelijke groeten,

Mede namens prof. Gansevoort en prof. de Fijter,

Emmelien Schillern, Damia Eleveld, Maya Levy en Lisanne Brenkman

----
Bachelor Project ADPKD & Nefrectomie
Afdeling Nefrologie
bp.adpkd@gmail.com
Voor vragen kunt u bellen naar:
050-3616161 (UMCG), vraag naar prof. Gansevoort, afdeling Nefrologie
071-5269111 (LUMC), vraag naar prof. de Fijter, afdeling Nierziekten

VRAGENLIJST ONDERZOEK NIERVERWIJDERING

Toelichting: Deze vragenlijst bestaat uit zes onderdelen, genummerd 1 tot en met 6. In deel 1 en 2 wordt gevraagd naar uw algemene gegevens en ADPKD(cystenieren)-gerelateerde medische voorgeschiedenis. Delen 3, 4 en 5 van deze vragenlijst bestaan uit 4 subonderdelen. Deze bevatten vragen over uw algemene gezondheid en specifieke klachten op een bepaald moment in uw leven, ten opzichte van de niertransplantatie die u destijds bent ondergaan. Op deze manier kunnen wij de klachten op deze momenten met elkaar vergelijken. Deel 3 vraagt naar uw gezondheidstoestand op dit moment, deel 4 naar uw gezondheidstoestand 12 maanden voor uw niertransplantatie en deel 5 naar uw gezondheidstoestand 12 maanden na uw transplantatie. Deel 6 gaat uiteindelijk over uw tevredenheid over de transplantatie en/of uw nierverwijdering.

We verzoeken u de vragen zo zorgvuldig mogelijk in te vullen. Als u al enkele jaren geleden bent getransplanteerd, kan het best moeilijk zijn om de vragenlijst in te vullen. We vragen hiervoor uw begrip. We adviseren u, als het kan, om de vragenlijst samen met uw partner, naaste familielid of een vriend(in) in te vullen. Mocht u het antwoord op één van de vragen in de vragenlijst niet weten, of mocht een vraag niet van toepassing voor u zijn, kunt u hier een streepje ('-') invullen. Om goede uitkomsten te krijgen voor het onderzoek, is het wel belangrijk dat u elke vraag invult in de delen B, C en D. Mocht u een vraag willen wijzigen, dan kunt u het best een horizontale streep door het incorrecte antwoorden tekenen en hierna het correcte antwoord invullen.

Overzicht

Deel 1 – Algemene gegevens 5

Deel 2 – ADPKD gerelateerde medische voorgeschiedenis 6

Deel 3 – Gezondheid op dit moment
- Deel 3A – ADPKD gerelateerde klachten *op dit moment* 9
- Deel 3B – SF-36 Gezondheidstoestand *op dit moment* 10
- Deel 3C – ADPKD Impact Scale *op dit moment* 13
- Deel 3D – Patient Health Questionnaire *op dit moment* 14

Deel 4 – Gezondheid 12 maanden *voor* uw transplantatie
- Deel 4A – ADPKD gerelateerde klachten *12 maanden voor uw transplantatie* 15
- Deel 4B – SF-36 Gezondheidstoestand *12 maanden voor uw transplantatie* 16
- Deel 4C – ADPKD Impact Scale *12 maanden voor uw transplantatie* 19
- Deel 4D – Patient Health Questionnaire *12 maanden voor uw transplantatie* 20

Deel 5 – Gezondheid 12 maanden *na* uw transplantatie
- Deel 5A – ADPKD gerelateerde klachten 12 maanden *na uw transplantatie* 21
- Deel 5B – SF-36 Gezondheidstoestand *12 maanden na uw transplantatie* 22
- Deel 5C – ADPKD Impact Scale *12 maanden na transplantatie* 25
- Deel 5D – Patient Health Questionnaire *12 maanden na uw transplantatie* 26

Deel 6 – Algemene tevredenheid ingreep 27

Deel 1. Algemene gegevens

1. In welk ziekenhuis bent u getransplanteerd?
   - ☐ Amsterdam MC
   - ☐ Radboud UMC
   - ☐ UMC Groningen
   - ☐ Leiden UMC
   - ☐ Erasmus MC
   - ☐ UMC Utrecht
   - ☐ VUMC Amsterdam
   - ☐ Maastricht UMC
2. Hebt u een nierverwijdering ondergaan? ☐ Ja ☐ Nee
3. Geslacht ☐ Man ☐ Vrouw
4. Lengte __________ cm
5. Gewicht __________ kg
6. Wat is de hoogste afleiding die u heeft afgerond?
   - ☐ Geen onderwijs
   - ☐ MBO
   - ☐ Basisonderwijs
   - ☐ HAVO/VWO
   - ☐ VMBO/MBO/LBO
   - ☐ HBO/WO
7. Wat is uw huidige werksituatie?
   - ☐ Ik heb een betaalde baan
   - ☐ Ik ben werkloos en werkzoekend
   - ☐ Ik heb geen betaalde baan, ik doe het huishouden
   - ☐ Ik heb geen baan, want ik ben arbeidsongeschikt
   - ☐ Ik ben met pensioen (of VUT) en had vroeger een betaalde baan
   - ☐ Ik ben ouder dan 65 en heb vroeger nooit een betaalde baan gehad
   - ☐ Anders
8. Werkt u minder, of doet u ander werk dan u zou willen, doordat u cystenieren heeft? ☐ Ja ☐ Nee
9. Sport u?
   - ☐ Nee, ik sport niet vanwege mijn cystenieren
   - ☐ Nee, ik sport niet vanwege een andere reden
   - ☐ Ja, meestal licht intensief (geen verhoogde hartslag/versnelde ademhaling)
   - ☐ Ja, meestal matig intensief (verhoogde ademhaling en versnelde ademhaling)
   - ☐ Ja, meestal zwaar intensief (waarbij u gaat zweten en buiten adem raakt)
10. Hoe vaak sport u per week? ________ keer
11. Hoe veel uur sport u totaal per week? _________ uur
12. Wat is uw gehuwde status
    - ☐ Alleenstaand
    - ☐ Gescheiden
    - ☐ Met partner
    - ☐ Geweduwd
13. Heeft u kinderen? ☐ Ja ☐ Nee

Deel 2. ADPKD-gerelateerde medische voorgeschiedenis

1. Jaar waarin cystenieren bij u zijn vastgesteld ____________
2. Datum niertransplantatie ______/______/____________
3. Heeft u vóór de transplantatie dialyse ondergaan? ☐ Ja ☐ Nee
   3a. Wat voor soort dialyse? ☐ Peritoneaal dialyse ☐ Hemodialyse
   3b. Maand en jaar start dialyse ______/____________
4. Heeft u ook cystes in de lever? ☐ Ja ☐ Nee
5. Heeft u naast cystenieren nog andere aandoeningen? (niet veroorzaakt door uw cystenieren)
   ☐ Nee
   ☐ Ja, diabetes
   ☐ Ja, hart en vaatziekte
   ☐ Ja, COPD
   ☐ Ja, kanker
   ☐ Ja, anders nl ___________________
6. Heeft u eerder een buikoperatie gehad? ☐ Ja ☐ Nee

Vul de volgende vragen alleen in als bij u <u>één of twee nieren</u> zijn verwijderd.

7. Hoeveel nier(en) is/zijn verwijderd? ☐ Eén ☐ Beide

8a Datum (eerste) nierverwijdering ______/______/____________

8b Welke nier is (eerst) verwijderd? ☐ Links ☐ Rechts ☐ Beide tegelijk

8c Op welke manier is de eerste nier verwijderd? ☐ Laparoscopisch ☐ Open

8d Heeft u een verticaal of horizontaal litteken? ☐ Verticaal ☐ Horizontaal

8e In welk ziekenhuis heeft u deze nier laten verwijderen?
☐ Amsterdam MC ☐ Radboud UMC
☐ UMC Groningen ☐ Leiden UMC
☐ Erasmus MC ☐ UMC Utrecht
☐ VUMC Amsterdam ☐ Maastricht UMC
☐ Anders, nl ________________________

8f Wat was de **belangrijkste** reden voor het verwijderen van deze nier? (er staan nog vier opties op de volgende pagina)
☐ Te weinig ruimte voor de nieuwe nier
☐ Terugkerende cyste infecties
☐ Terugkerende blaasontsteking
☐ Nierstenen (met of zonder ontstekingen)
☐ Onbehandelbare pijn
☐ Bloed in de urine
☐ Snel vol gevoel na eten
☐ Slecht verdragen van eten
☐ Obstipatie

☐ Gewichtsverlies
☐ Eigen voorkeur
☐ Weet ik niet
☐ Andere reden, nl ____________________

8g	Is de eerste nier voor, na, of tijdens de transplantatie verwijderd?	☐ Voor ☐ Na ☐ Tijdens
8h	Hoe zwaar was de eerste nier (ongeveer)?	__________ kg

Vul de volgende vragen alleen in als bij u <u>een tweede nier</u> is verwijderd.

9a	Datum tweede nierverwijdering	______/______/____________
9b	Op welke manier werd die nier verwijderd?	☐ Laparoscopisch ☐ Open
9c	Heeft u een verticaal of horizontaal litteken?	☐ Verticaal ☐ Horizontaal
9d	In welk ziekenhuis heeft u deze nier laten verwijderen?	☐ Amsterdam MC ☐ Radboud UMC<br>☐ UMC Groningen ☐ Leiden UMC<br>☐ Erasmus MC ☐ UMC Utrecht<br>☐ VUMC Amsterdam ☐ Maastricht UMC<br>☐ Anders, nl ________________________
9e	Wat was de **belangrijkste** reden voor het verwijderen van deze nier?	☐ Te weinig ruimte voor de nieuwe nier<br>☐ Terugkerende cyste infecties<br>☐ Terugkerende blaasontstekingen<br>☐ Nierstenen (met of zonder ontstekingen)<br>☐ Onbehandelbare pijn<br>☐ Bloed in de urine<br>☐ Snel vol gevoel na eten<br>☐ Slecht verdragen van eten<br>☐ Obstipatie<br>☐ Gewichtsverlies<br>☐ Eigen voorkeur<br>☐ Weet ik niet<br>☐ Andere reden, nl ____________________
9f	Is de tweede nier voor, na, of tijdens de transplantatie verwijderd?	☐ Voor ☐ Na ☐ Tijdens
9h	Hoe zwaar was de tweede nier (ongeveer)?	__________ kg

Deel 3: Gezondheid ***op dit moment***

Deel 3A: ADPKD-gerelateerde klachten ***op dit moment***

1. Hoeveel last had u de afgelopen twee weken van de volgende klachten?

	Helemaal niet	Een beetje	Nogal	Veel	Heel erg veel
Drukkend gevoel in de buik	☐	☐	☐	☐	☐
Slecht verdragen van eten	☐	☐	☐	☐	☐
Snel vol gevoel na eten	☐	☐	☐	☐	☐
Weinig eetlust	☐	☐	☐	☐	☐
Gewichtsverlies	☐	☐	☐	☐	☐
Misselijkheid	☐	☐	☐	☐	☐
Overgeven	☐	☐	☐	☐	☐
Maagzuurklachten	☐	☐	☐	☐	☐
Obstipatie	☐	☐	☐	☐	☐
Vermoeidheid	☐	☐	☐	☐	☐
Nier-gerelateerde pijn	☐	☐	☐	☐	☐
Lever-gerelateerde pijn	☐	☐	☐	☐	☐
Bloed in de urine	☐	☐	☐	☐	☐
Blaasontsteking	☐	☐	☐	☐	☐
Nierstenen	☐	☐	☐	☐	☐
Anders, namelijk: ______________________	☐	☐	☐	☐	☐

Deel 3B: SF-36 Gezondheidstoestand *op dit moment*

1. Hoe zou u over het algemeen uw gezondheid noemen?

- ☐ Uitstekend
- ☐ Zeer goed
- ☐ Goed
- ☐ Matig
- ☐ Slecht

2. Hoe beoordeelt u nu uw gezondheid over het algemeen, vergeleken met een jaar geleden?

- ☐ Veel beter dan een jaar geleden
- ☐ Een beetje beter dan een jaar geleden
- ☐ Ongeveer hetzelfde als een jaar geleden
- ☐ Een beetje slechter dan een jaar geleden
- ☐ Veel slechter dan een jaar geleden

3. De volgende vragen gaan over bezigheden die u misschien doet op een doorsnee dag. Wordt u door uw gezondheid op dit moment beperkt bij deze bezigheden? Zo ja, in welke mate?

	Ja, ernstig beperkt	Ja, een beetje beperkt	Nee, helemaal niet beperkt
a. Forse inspanning, zoals hardlopen, tillen van zware voorwerpen, een veeleisende sport beoefenen	☐	☐	☐
b. Matige inspanning, zoals een tafel verplaatsen, stofzuigen, zwemmen of fietsen	☐	☐	☐
c. Boodschappen tillen of dragen	☐	☐	☐
d. Een paar trappen oplopen	☐	☐	☐
e. Eén trap oplopen	☐	☐	☐
f. Bukken, knielen of hurken	☐	☐	☐
g. Meer dan een kilometer lopen	☐	☐	☐
h. Een paar honderd meter lopen	☐	☐	☐
i. Ongeveer honderd meter lopen	☐	☐	☐
j. Uzelf wassen of aankleden	☐	☐	☐

4. Heeft u in de afgelopen vier weken één van de volgende problemen bij uw werk of andere dagelijkse bezigheden gehad, vanwege uw lichamelijke gezondheid?

	Ja	Nee
a. U besteedde minder tijd aan werk of andere bezigheden	☐	☐
b. U heeft minder bereikt dan u zou willen	☐	☐
c. U was beperkt in het soort werk of andere bezigheden	☐	☐
d. U had moeite om uw werk of andere bezigheden uit te voeren (het kostte u bv. extra inspanning)	☐	☐

5. Heeft u in de afgelopen vier weken één van de volgende problemen ondervonden bij uw werk of andere dagelijkse bezigheden ten gevolge van emotionele problemen (zoals depressieve of angstige gevoelens)?

	Ja	Nee
a. U besteedde minder tijd aan werk of andere bezigheden	☐	☐
b. U heeft minder bereikt dan u zou willen	☐	☐
c. U deed uw werk of andere bezigheden niet zo zorgvuldig als gewoonlijk	☐	☐

6. In hoeverre hebben uw lichamelijke gezondheid of emotionele problemen u gedurende de afgelopen vier weken gehinderd in uw normale omgang met familie, vrienden of buren, of bij activiteiten in groepsverband?

- ☐ Helemaal niet
- ☐ Enigszins
- ☐ Nogal
- ☐ Veel
- ☐ Heel erg veel

7. Hoeveel lichamelijke pijn heeft u de afgelopen vier weken gehad?

- ☐ Geen
- ☐ Heel licht
- ☐ Licht
- ☐ Nogal
- ☐ Ernstig
- ☐ Heel ernstig

8. In welke mate bent u de afgelopen vier weken door pijn gehinderd in uw normale werk (zowel werk buitenshuis als huishoudelijk werk)?

- ☐ Helemaal niet
- ☐ Een klein beetje
- ☐ Nogal
- ☐ Veel
- ☐ Heel erg veel

9. Deze vragen gaan over hoe u zich voelt en hoe het met u ging in de afgelopen vier weken. Wilt u a.u.b. bij elke vraag het antwoord geven dat het best benadert hoe u zich voelde. Hoe vaak gedurende de afgelopen vier weken ... (vink één hokje aan op elke regel)

	Altijd	Meestal	Vaak	Soms	Zelden	Nooit
a. Voelde u zich levenslustig?	☐	☐	☐	☐	☐	☐
b. Was u erg zenuwachtig?	☐	☐	☐	☐	☐	☐
c. Zat u zo in de put dat niets u kon opvrolijken?	☐	☐	☐	☐	☐	☐
d. Voelde u zich rustig en tevreden?	☐	☐	☐	☐	☐	☐
e. Had u veel energie?	☐	☐	☐	☐	☐	☐
f. Voelde u zich somber en neerslachtig?	☐	☐	☐	☐	☐	☐
g. Voelde u zich uitgeput?	☐	☐	☐	☐	☐	☐
h. Was u een gelukkig mens?	☐	☐	☐	☐	☐	☐
i. Voelde u zich moe?	☐	☐	☐	☐	☐	☐

10. Hoe vaak hebben uw lichamelijke gezondheid of emotionele problemen u gedurende de afgelopen vier weken gehinderd bij uw sociale activiteiten (vrienden of familie bezoeken etc)?

☐ Altijd
☐ Meestal
☐ Soms
☐ Zelden
☐ Nooit

11. Hoe JUIST of ONJUIST is elk van de volgende uitspraken voor u?

	volkomen juist	grotendeels juist	weet ik niet	grotendeels onjuist	volkomen onjuist
a. Ik lijk wat gemakkelijker ziek te worden	☐	☐	☐	☐	☐
b. Ik ben even gezond als andere mensen	☐	☐	☐	☐	☐
c. Ik verwacht dat mijn gezondheid achteruit zal gaan	☐	☐	☐	☐	☐
d. Mijn gezondheid is uitstekend	☐	☐	☐	☐	☐

Deel 3C: ADPKD Impact Scale ***op dit moment***

1. Als het gaat over cystenieren (ADPKD), hoe moeilijk was het voor u in de afgelopen twee weken om...

	Helemaal niet moeilijk	Een beetje moeilijk	Nogal moeilijk	Erg moeilijk	Uiterst moeilijk
a. Vrijetijdsactiviteiten en lichte oefeningen uit te voeren, zoals tuinieren, lopen of rekoefeningen?	☐	☐	☐	☐	☐
b. Een hele dag werk in uw baan of thuis te voltooien (huishoudelijk werk, zoals stofzuigen of de was doen)?	☐	☐	☐	☐	☐
c. Dagelijkse activiteiten zoals gewoonlijk uit te voeren, ongeacht de pijn die u hebt ervaren door uw ADPKD?	☐	☐	☐	☐	☐
d. Alles af te maken wat u op een dag wilde doen, omdat u zich moe of uitgeput voelde?	☐	☐	☐	☐	☐
e. Zwaardere lichamelijke activiteiten uit te voeren, zoals tillen, hardlopen of sporten?	☐	☐	☐	☐	☐
f. ADPKD als een deel van uw leven te aanvaarden?	☐	☐	☐	☐	☐
g. Om te gaan met schuldgevoel over de invloed van uw ADPKD op uw kinderen of andere familieleden?	☐	☐	☐	☐	☐
h. Te slapen vanwege uw ADPKD?	☐	☐	☐	☐	☐

2. Als het gaat over ADPKD, hoeveel hinder had u in de afgelopen twee weken van...

	Helemaal geen hinder	Een beetje hinder	Nogal hinder	Heel veel hinder	Erg veel hinder
a. De omvang en/of vorm van uw buik?	☐	☐	☐	☐	☐
b. U uitgeput of vermoeid voelen?	☐	☐	☐	☐	☐
c. U angstig voelen omdat u weet dat uw ADPKD erger kan worden?	☐	☐	☐	☐	☐
d. U verdrietig voelen omdat u ADPKD hebt?	☐	☐	☐	☐	☐
e. U vol voelen voordat uw eetlust is gestild?	☐	☐	☐	☐	☐
f. Vaker en/of dringend te moeten urineren?	☐	☐	☐	☐	☐
g. Uw levensstijl te moeten wijzigen (zoals de manier waarop u slaapt of lichaamsbeweging beperken) vanwege ongemak of pijn door uw ADPKD?	☐	☐	☐	☐	☐
h. Uw pijn in verband met ADPKD?	☐	☐	☐	☐	☐
i. U moe voelen op weg van/naar school of werk of als u boodschappen doet?	☐	☐	☐	☐	☐
j. U de volgende dag vermoeid voelen, zelfs na een nacht slapen?	☐	☐	☐	☐	☐

Deel 3D: Patient Health Questionnaire *op dit moment*

Hoe vaak hebt u in de afgelopen twee weken last gehad van één of meer van de volgende problemen?

		Helemaal niet	Verscheidene dagen	Meer dan de helft van de dagen	Bijna elke dag
1.	Weinig interesse of plezier om dingen te doen	☐	☐	☐	☐
2.	Zich neerslachtig, gedeprimeerd of hopeloos voelen	☐	☐	☐	☐
3.	Moeilijk inslapen, moeilijk doorslapen of teveel slapen	☐	☐	☐	☐
4.	Zich moe voelen of gebrek aan energie hebben	☐	☐	☐	☐
5.	Weinig eetlust of overmatig eten	☐	☐	☐	☐
6.	Een slecht gevoel hebben over uzelf of het gevoel hebben dat u een mislukking bent of het gevoel dat u zichzelf of uw familie teleurgesteld hebt	☐	☐	☐	☐
7.	Problemen om u te concentreren, bijvoorbeeld om de krant te lezen of om tv te kijken	☐	☐	☐	☐
8.	Zo traag bewegen of zo langzaam spreken dat andere mensen dit opgemerkt kunnen hebben? Of het tegenovergestelde, zo zenuwachtig of rusteloos zijn dat u veel meer bewoog dan gebruikelijk	☐	☐	☐	☐
9.	De gedachte dat u beter dood zou kunnen zijn of de gedachte uzelf op een bepaalde manier pijn te doen	☐	☐	☐	☐

10. Als u **enig** probleem hebt aangekruist, hoe **moeilijk** maakten deze problemen het dan voor u om uw werk of uw taken in en om het huis te doen, of om met andere mensen om te gaan?

☐ Helemaal niet moeilijk ☐ Enigszins moeilijk ☐ Erg moeilijk ☐ Extreem moeilijk

Deel 4. Gezondheid _**12 maanden voor uw transplantatie**_

Toelichting: Probeert u bij het invullen van dit onderdeel terug te denken aan hoe u zich in de periode van 12 tot 13 maanden voor de transplantatie voelde. Hoe zag uw leven er toen uit? Van wat voor klachten had u vooral last? Waar had u moeite mee en wat kon u juist nog wel goed doen? We adviseren u, indien mogelijk, om uw partner, naaste familielid of een vriend(in) te betrekken bij het invullen. Indien uw niertransplantatie al enkele jaren geleden heeft plaatsgevonden kan het goed invullen van de vragen lastig zijn. We vragen hiervoor uw begrip.

Deel 4A: ADPKD-gerelateerde klachten _**12 maanden voor uw transplantatie**_

1. Hoeveel last had u 12 maanden voor uw transplantatie van de volgende klachten?

	Helemaal niet	Een beetje	Nogal	Veel	Heel erg veel
Drukkend gevoel in de buik	☐	☐	☐	☐	☐
Slecht verdragen van eten	☐	☐	☐	☐	☐
Snel vol gevoel na eten	☐	☐	☐	☐	☐
Weinig eetlust	☐	☐	☐	☐	☐
Gewichtsverlies	☐	☐	☐	☐	☐
Misselijkheid	☐	☐	☐	☐	☐
Overgeven	☐	☐	☐	☐	☐
Maagzuurklachten	☐	☐	☐	☐	☐
Obstipatie	☐	☐	☐	☐	☐
Vermoeidheid	☐	☐	☐	☐	☐
Nier-gerelateerde pijn	☐	☐	☐	☐	☐
Lever-gerelateerde pijn	☐	☐	☐	☐	☐
Bloed in de urine	☐	☐	☐	☐	☐
Terugkerende blaasontstekingen	☐	☐	☐	☐	☐
Nierstenen	☐	☐	☐	☐	☐
Anders, namelijk: ________________________	☐	☐	☐	☐	☐

Deel 4B: SF-36 Gezondheidstoestand *12 maanden voor uw transplantatie*

1. Hoe zou u over het algemeen uw gezondheid noemen?

- ☐ Uitstekend
- ☐ Zeer goed
- ☐ Goed
- ☐ Matig
- ☐ Slecht

2. Hoe beoordeelde u uw gezondheid over het algemeen, vergeleken met een jaar daarvoor?

- ☐ Veel beter dan een jaar daarvoor
- ☐ Iets beter dan een jaar daarvoor
- ☐ Ongeveer hetzelfde als een jaar daarvoor
- ☐ Wat slechter dan een jaar daarvoor
- ☐ Veel slechter dan een jaar daarvoor

3. De volgende vragen gaan over bezigheden die u misschien doet op een doorsnee dag. Werd u door uw gezondheid 12 maanden voor uw transplantatie beperkt bij deze bezigheden? Zo ja, in welke mate?

	Ja, ernstig beperkt	Ja, een beetje beperkt	Nee, helemaal niet beperkt
a. Forse inspanning, zoals hardlopen, tillen van zware voorwerpen, een veeleisende sport beoefenen	☐	☐	☐
b. Matige inspanning, zoals een tafel verplaatsen, stofzuigen, zwemmen of fietsen	☐	☐	☐
c. Boodschappen tillen of dragen	☐	☐	☐
d. Een paar trappen oplopen	☐	☐	☐
e. Eén trap oplopen	☐	☐	☐
f. Bukken, knielen of hurken	☐	☐	☐
g. Meer dan een kilometer lopen	☐	☐	☐
h. Een paar honderd meter lopen	☐	☐	☐
i. Ongeveer honderd meter lopen	☐	☐	☐
j. Uzelf wassen of aankleden	☐	☐	☐

4. Had u 12 maanden voor uw transplantatie één van de volgende problemen bij uw werk of andere dagelijkse bezigheden gehad, vanwege uw lichamelijke gezondheid?

	Ja	Nee
a. U besteedde minder tijd aan werk of andere bezigheden	☐	☐
b. U had minder bereikt dan u zou willen	☐	☐
c. U was beperkt in het soort werk of andere bezigheden	☐	☐
d. U had moeite om uw werk of andere bezigheden uit te voeren (het kostte u bv. extra inspanning)	☐	☐

5. Had u 12 maanden voor uw transplantatie één van de volgende problemen ondervonden bij uw werk of andere dagelijkse bezigheden ten gevolge van emotionele problemen (zoals depressieve of angstige gevoelens)?

	Ja	Nee
a. U besteedde minder tijd aan werk of andere bezigheden	☐	☐
b. U had minder bereikt dan u zou willen	☐	☐
c. U deed uw werk of andere bezigheden niet zo zorgvuldig als gewoonlijk	☐	☐

6. In hoeverre hadden uw lichamelijke gezondheid of emotionele problemen u 12 maanden voor uw transplantatie gehinderd in uw normale omgang met familie, vrienden of buren, of bij activiteiten in groepsverband?

- ☐ Helemaal niet
- ☐ Enigszins
- ☐ Nogal
- ☐ Veel
- ☐ Heel erg veel

7. Hoeveel lichamelijke pijn had u 12 maanden voor uw transplantatie?

- ☐ Geen
- ☐ Heel licht
- ☐ Licht
- ☐ Nogal
- ☐ Ernstig
- ☐ Heel ernstig

8. In welke mate was u 12 maanden voor uw transplantatie door pijn gehinderd in uw normale werk (zowel werk buitenshuis als huishoudelijk werk)?

- ☐ Helemaal niet
- ☐ Een klein beetje
- ☐ Nogal
- ☐ Veel
- ☐ Heel erg veel

9. Deze vragen gaan over hoe u zich voelde en hoe het met u ging 12 maanden voor uw transplantatie. Wilt u a.u.b. bij elke vraag het antwoord geven dat het best benadert hoe u zich voelde. Hoe vaak gedurende de periode van 12-13 maanden voor uw transplantatie ... (vink één hokje aan op elke regel)

	Altijd	Meestal	Vaak	Soms	Zelden	Nooit
a. Voelde u zich levenslustig?	☐	☐	☐	☐	☐	☐
b. Was u erg zenuwachtig?	☐	☐	☐	☐	☐	☐
c. Zat u zo in de put dat niets u kon opvrolijken?	☐	☐	☐	☐	☐	☐
d. Voelde u zich rustig en tevreden?	☐	☐	☐	☐	☐	☐
e. Had u veel energie?	☐	☐	☐	☐	☐	☐
f. Voelde u zich somber en neerslachtig?	☐	☐	☐	☐	☐	☐
g. Voelde u zich uitgeput?	☐	☐	☐	☐	☐	☐
h. Was u een gelukkig mens?	☐	☐	☐	☐	☐	☐
i. Voelde u zich moe?	☐	☐	☐	☐	☐	☐

10. Hoe vaak hadden uw lichamelijke gezondheid of emotionele problemen u gehinderd bij uw sociale activiteiten (vrienden of familie bezoeken etc)?

☐ Altijd
☐ Meestal
☐ Soms
☐ Zelden
☐ Nooit

11. Hoe JUIST of ONJUIST is elk van de volgende uitspraken voor u?

	volkomen juist	grotendeels juist	weet ik niet	grotendeels onjuist	volkomen onjuist
a. Ik leek wat gemakkelijker ziek te worden	☐	☐	☐	☐	☐
b. Ik was even gezond als andere mensen	☐	☐	☐	☐	☐
c. Ik verwachtte dat mijn gezondheid achteruit zal gaan	☐	☐	☐	☐	☐
d. Mijn gezondheid was uitstekend	☐	☐	☐	☐	☐

Deel 4C: ADPKD Impact Scale *12 maanden voor uw transplantatie*

1. Als het gaat over cystenieren (ADPKD), hoe moeilijk was het voor u 12 maanden voor uw transplantatie om...

	Helemaal niet moeilijk	Een beetje moeilijk	Nogal moeilijk	Erg moeilijk	Uiterst moeilijk
a. Vrijetijdsactiviteiten en lichte oefeningen uit te voeren, zoals tuinieren, lopen of rekoefeningen?	☐	☐	☐	☐	☐
b. Een hele dag werk in uw baan of thuis te voltooien (huishoudelijk werk, zoals stofzuigen of de was doen)?	☐	☐	☐	☐	☐
c. Dagelijkse activiteiten zoals gewoonlijk uit te voeren, ongeacht de pijn die u hebt ervaren door uw ADPKD?	☐	☐	☐	☐	☐
d. Alles af te maken wat u op een dag wilde doen, omdat u zich moe of uitgeput voelde?	☐	☐	☐	☐	☐
e. Zwaardere lichamelijke activiteiten uit te voeren, zoals tillen, hardlopen of sporten?	☐	☐	☐	☐	☐
f. ADPKD als een deel van uw leven te aanvaarden?	☐	☐	☐	☐	☐
g. Om te gaan met schuldgevoel over de invloed van uw ADPKD op uw kinderen of andere familieleden?	☐	☐	☐	☐	☐
h. Te slapen vanwege uw ADPKD?	☐	☐	☐	☐	☐

2. Als het gaat over ADPKD, hoeveel hinder had u 12 maanden voor uw transplantatie van...

	Helemaal geen hinder	Een beetje hinder	Nogal hinder	Heel veel hinder	Erg veel hinder
a. De omvang en/of vorm van uw buik?	☐	☐	☐	☐	☐
b. U uitgeput of vermoeid voelen?	☐	☐	☐	☐	☐
c. U angstig voelen omdat u weet dat uw ADPKD erger kan worden?	☐	☐	☐	☐	☐
d. U verdrietig voelen omdat u ADPKD hebt?	☐	☐	☐	☐	☐
e. U vol voelen voordat uw eetlust is gestild?	☐	☐	☐	☐	☐
f. Vaker en/of dringend te moeten urineren?	☐	☐	☐	☐	☐
g. Uw levensstijl te moeten wijzigen (zoals de manier waarop u slaapt of lichaamsbeweging beperken) vanwege ongemak of pijn door uw ADPKD?	☐	☐	☐	☐	☐
h. Uw pijn in verband met ADPKD?	☐	☐	☐	☐	☐
i. U moe voelen op weg van/naar school of werk of als u boodschappen doet?	☐	☐	☐	☐	☐
j. U de volgende dag vermoeid voelen, zelfs na een nacht slapen?	☐	☐	☐	☐	☐

Deel 4D: Patient Health Questionnaire *12 maanden voor uw transplantatie*

Hoe vaak had u 12 maanden voor uw transplantatie last van één of meer van de volgende problemen?

		Helemaal niet	Verscheidene dagen	Meer dan de helft van de dagen	Bijna elke dag
1.	Weinig interesse of plezier om dingen te doen	☐	☐	☐	☐
2.	Zich neerslachtig, gedeprimeerd of hopeloos voelen	☐	☐	☐	☐
3.	Moeilijk inslapen, moeilijk doorslapen of teveel slapen	☐	☐	☐	☐
4.	Zich moe voelen of gebrek aan energie hebben	☐	☐	☐	☐
5.	Weinig eetlust of overmatig eten	☐	☐	☐	☐
6.	Een slecht gevoel hebben over uzelf of het gevoel hebben dat u een mislukking bent of het gevoel dat u zichzelf of uw familie teleurgesteld hebt	☐	☐	☐	☐
7.	Problemen om u te concentreren, bijvoorbeeld om de krant te lezen of om tv te kijken	☐	☐	☐	☐
8.	Zo traag bewegen of zo langzaam spreken dat andere mensen dit opgemerkt kunnen hebben? Of het tegenovergestelde, zo zenuwachtig of rusteloos zijn dat u veel meer bewoog dan gebruikelijk	☐	☐	☐	☐
9.	De gedachte dat u beter dood zou kunnen zijn of de gedachte uzelf op een bepaalde manier pijn te doen	☐	☐	☐	☐

10. Als u **enig** probleem hebt aangekruist, hoe **moeilijk** maakten deze problemen het dan voor u om uw werk of uw taken in en om het huis te doen, of om met andere mensen om te gaan?

☐ Helemaal niet moeilijk ☐ Enigszins moeilijk ☐ Erg moeilijk ☐ Extreem moeilijk

Deel 5: Gezondheid _12 maanden na uw transplantatie_

Toelichting: Probeert u bij het invullen van dit onderdeel terug te denken aan hoe u zich in de periode van 12 tot 13 maanden na uw transplantatie voelde. Hoe zag uw leven er toen uit? Van wat voor klachten had u vooral last? Waar had u moeite mee en wat kon u juist nog wel goed doen? We adviseren u, indien mogelijk, om uw partner, naaste familielid of een vriend(in) te betrekken bij het invullen. Indien uw niertransplantatie al enkele jaren geleden heeft plaatsgevonden kan het goed invullen van de vragen lastig zijn. We vragen hiervoor uw begrip.

Deel 5A: ADPKD-gerelateerde klachten _12 maanden na uw transplantatie_

1. Hoeveel last had u 12 maanden na uw transplantatie van de volgende klachten?

	Helemaal niet	Een beetje	Nogal	Veel	Heel erg veel
Drukkend gevoel in de buik	☐	☐	☐	☐	☐
Slecht verdragen van eten	☐	☐	☐	☐	☐
Snel vol gevoel na eten	☐	☐	☐	☐	☐
Weinig eetlust	☐	☐	☐	☐	☐
Gewichtsverlies	☐	☐	☐	☐	☐
Misselijkheid	☐	☐	☐	☐	☐
Overgeven	☐	☐	☐	☐	☐
Maagzuurklachten	☐	☐	☐	☐	☐
Obstipatie	☐	☐	☐	☐	☐
Vermoeidheid	☐	☐	☐	☐	☐
Nier-gerelateerde pijn	☐	☐	☐	☐	☐
Lever-gerelateerde pijn	☐	☐	☐	☐	☐
Bloed in de urine	☐	☐	☐	☐	☐
Terugkerende blaasontstekingen	☐	☐	☐	☐	☐
Nierstenen	☐	☐	☐	☐	☐
Anders, namelijk: ________________________	☐	☐	☐	☐	☐

Deel 5B: SF-36 Gezondheidstoestand ***12 maanden na de transplantatie***

1. Hoe zou u over het algemeen uw gezondheid noemen?

- ☐ Uitstekend
- ☐ Zeer goed
- ☐ Goed
- ☐ Matig
- ☐ Slecht

2. Hoe beoordeelde u uw gezondheid over het algemeen, vergeleken met een jaar daarvoor?

- ☐ Veel beter dan een jaar daarvoor
- ☐ Iets beter dan een jaar daarvoor
- ☐ Ongeveer hetzelfde als een jaar daarvoor
- ☐ Wat slechter dan een jaar daarvoor
- ☐ Veel slechter dan een jaar daarvoor

3. De volgende vragen gaan over bezigheden die u misschien doet op een doorsnee dag. Werd u door uw gezondheid 12 maanden na uw transplantatie beperkt bij deze bezigheden? Zo ja, in welke mate?

	Ja, ernstig beperkt	Ja, een beetje beperkt	Nee, helemaal niet beperkt
a. Forse inspanning, zoals hardlopen, tillen van zware voorwerpen, een veeleisende sport beoefenen	☐	☐	☐
b. Matige inspanning, zoals een tafel verplaatsen, stofzuigen, zwemmen of fietsen	☐	☐	☐
c. Boodschappen tillen of dragen	☐	☐	☐
d. Een paar trappen oplopen	☐	☐	☐
e. Eén trap oplopen	☐	☐	☐
f. Bukken, knielen of hurken	☐	☐	☐
g. Meer dan een kilometer lopen	☐	☐	☐
h. Een paar honderd meter lopen	☐	☐	☐
i. Ongeveer honderd meter lopen	☐	☐	☐
j. Uzelf wassen of aankleden	☐	☐	☐

4. Had u 12 maanden na uw transplantatie één van de volgende problemen bij uw werk of andere dagelijkse bezigheden gehad, vanwege uw lichamelijke gezondheid?

	Ja	Nee
a. U besteedde minder tijd aan werk of andere bezigheden	☐	☐
b. U had minder bereikt dan u zou willen	☐	☐
c. U was beperkt in het soort werk of andere bezigheden	☐	☐
d. U had moeite om uw werk of andere bezigheden uit te voeren (het kostte u bv. extra inspanning)	☐	☐

5. Had u 12 maanden na uw transplantatie één van de volgende problemen ondervonden bij uw werk of andere dagelijkse bezigheden ten gevolge van emotionele problemen (zoals depressieve of angstige gevoelens)?

	Ja	Nee
a. U besteedde minder tijd aan werk of andere bezigheden	☐	☐
b. U had minder bereikt dan u zou willen	☐	☐
c. U deed uw werk of andere bezigheden niet zo zorgvuldig als gewoonlijk	☐	☐

6. In hoeverre hadden uw lichamelijke gezondheid of emotionele problemen u 12 maanden na uw transplantatie gehinderd in uw normale omgang met familie, vrienden of buren, of bij activiteiten in groepsverband?

- ☐ Helemaal niet
- ☐ Enigszins
- ☐ Nogal
- ☐ Veel
- ☐ Heel erg veel

7. Hoeveel lichamelijke pijn had u 12 maanden na uw transplantatie?

- ☐ Geen
- ☐ Heel licht
- ☐ Licht
- ☐ Nogal
- ☐ Ernstig
- ☐ Heel ernstig

8. In welke mate was u 12 maanden na uw transplantatie door pijn gehinderd in uw normale werk (zowel werk buitenshuis als huishoudelijk werk)?

- ☐ Helemaal niet
- ☐ Een klein beetje
- ☐ Nogal
- ☐ Veel
- ☐ Heel erg veel

9. **Deze vragen gaan over hoe u zich voelde en hoe het met u ging 12 maanden na uw transplantatie. Wilt u a.u.b. bij elke vraag het antwoord geven dat het best benadert hoe u zich voelde. Hoe vaak gedurende de periode van 12-13 maanden na uw transplantatie ... (vink één hokje aan op elke regel)**

	Altijd	Meestal	Vaak	Soms	Zelden	Nooit
a. Voelde u zich levenslustig?	☐	☐	☐	☐	☐	☐
b. Was u erg zenuwachtig?	☐	☐	☐	☐	☐	☐
c. Zat u zo in de put dat niets u kon opvrolijken?	☐	☐	☐	☐	☐	☐
d. Voelde u zich rustig en tevreden?	☐	☐	☐	☐	☐	☐
e. Had u veel energie?	☐	☐	☐	☐	☐	☐
f. Voelde u zich somber en neerslachtig?	☐	☐	☐	☐	☐	☐
g. Voelde u zich uitgeput?	☐	☐	☐	☐	☐	☐
h. Was u een gelukkig mens?	☐	☐	☐	☐	☐	☐
i. Voelde u zich moe?	☐	☐	☐	☐	☐	☐

10. **Hoe vaak hadden uw lichamelijke gezondheid of emotionele problemen u 12 maanden na uw transplantatie gehinderd bij uw sociale activiteiten (vrienden of familie bezoeken etc)?**

- ☐ Altijd
- ☐ Meestal
- ☐ Soms
- ☐ Zelden
- ☐ Nooit

11. **Hoe JUIST of ONJUIST is elk van de volgende uitspraken voor u?**

	volkomen juist	grotendeels juist	weet ik niet	grotendeels onjuist	volkomen onjuist
a. Ik leek wat gemakkelijker ziek te worden	☐	☐	☐	☐	☐
b. Ik was even gezond als andere mensen	☐	☐	☐	☐	☐
c. Ik verwachtte dat mijn gezondheid achteruit zal gaan	☐	☐	☐	☐	☐
d. Mijn gezondheid was uitstekend	☐	☐	☐	☐	☐

Deel 5C: ADPKD Impact Scale *12 maanden na uw transplantatie*

1. Als het gaat over cystenieren (ADPKD), hoe moeilijk was het voor u 12 maanden na uw transplantatie om...

	Helemaal niet moeilijk	Een beetje moeilijk	Nogal moeilijk	Erg moeilijk	Uiterst moeilijk
a. Vrijetijdsactiviteiten en lichte oefeningen uit te voeren, zoals tuinieren, lopen of rekoefeningen?	☐	☐	☐	☐	☐
b. Een hele dag werk in uw baan of thuis te voltooien (huishoudelijk werk, zoals stofzuigen of de was doen)?	☐	☐	☐	☐	☐
c. Dagelijkse activiteiten zoals gewoonlijk uit te voeren, ongeacht de pijn die u hebt ervaren door uw ADPKD?	☐	☐	☐	☐	☐
d. Alles af te maken wat u op een dag wilde doen, omdat u zich moe of uitgeput voelde?	☐	☐	☐	☐	☐
e. Zwaardere lichamelijke activiteiten uit te voeren, zoals tillen, hardlopen of sporten?	☐	☐	☐	☐	☐
f. ADPKD als een deel van uw leven te aanvaarden?	☐	☐	☐	☐	☐
g. Om te gaan met schuldgevoel over de invloed van uw ADPKD op uw kinderen of andere familieleden?	☐	☐	☐	☐	☐
h. Te slapen vanwege uw ADPKD?	☐	☐	☐	☐	☐

2. Als het gaat over ADPKD, hoeveel hinder had u 12 maanden na uw transplantatie van...

	Helemaal geen hinder	Een beetje hinder	Nogal hinder	Heel veel hinder	Erg veel hinder
a. De omvang en/of vorm van uw buik?	☐	☐	☐	☐	☐
b. U uitgeput of vermoeid voelen?	☐	☐	☐	☐	☐
c. U angstig voelen omdat u weet dat uw ADPKD erger kan worden?	☐	☐	☐	☐	☐
d. U verdrietig voelen omdat u ADPKD hebt?	☐	☐	☐	☐	☐
e. U vol voelen voordat uw eetlust is gestild?	☐	☐	☐	☐	☐
f. Vaker en/of dringend te moeten urineren?	☐	☐	☐	☐	☐
g. Uw levensstijl te moeten wijzigen (zoals de manier waarop u slaapt of lichaamsbeweging beperken) vanwege ongemak of pijn door uw ADPKD?	☐	☐	☐	☐	☐
h. Uw pijn in verband met ADPKD?	☐	☐	☐	☐	☐
i. U moe voelen op weg van/naar school of werk of als u boodschappen doet?	☐	☐	☐	☐	☐
j. U de volgende dag vermoeid voelen, zelfs na een nacht slapen?	☐	☐	☐	☐	☐

Deel 5D: Patient Health Questionnaire ***12 maanden na uw transplantatie***

Hoe vaak had u 12 maanden na uw transplantatie last van één of meer van de volgende problemen?

		Helemaal niet	Verscheidene dagen	Meer dan de helft van de dagen	Bijna elke dag
1.	Weinig interesse of plezier om dingen te doen	☐	☐	☐	☐
2.	Zich neerslachtig, gedeprimeerd of hopeloos voelen	☐	☐	☐	☐
3.	Moeilijk inslapen, moeilijk doorslapen of teveel slapen	☐	☐	☐	☐
4.	Zich moe voelen of gebrek aan energie hebben	☐	☐	☐	☐
5.	Weinig eetlust of overmatig eten	☐	☐	☐	☐
6.	Een slecht gevoel hebben over uzelf of het gevoel hebben dat u een mislukking bent of het gevoel dat u zichzelf of uw familie teleurgesteld hebt	☐	☐	☐	☐
7.	Problemen om u te concentreren, bijvoorbeeld om de krant te lezen of om tv te kijken	☐	☐	☐	☐
8.	Zo traag bewegen of zo langzaam spreken dat andere mensen dit opgemerkt kunnen hebben? Of het tegenovergestelde, zo zenuwachtig of rusteloos zijn dat u veel meer bewoog dan gebruikelijk	☐	☐	☐	☐
9.	De gedachte dat u beter dood zou kunnen zijn of de gedachte uzelf op een bepaalde manier pijn te doen	☐	☐	☐	☐

10. Als u **enig** probleem hebt aangekruist, hoe **moeilijk** maakten deze problemen het dan voor u om uw werk of uw taken in en om het huis te doen, of om met andere mensen om te gaan?

☐ Helemaal niet moeilijk ☐ Enigszins moeilijk ☐ Erg moeilijk ☐ Extreem moeilijk

Deel 6: Algemene tevredenheid ingreep

Vul vraag 1 in wanneer u geen nierverwijdering heeft ondergaan.

		Ja	Nee
**1.** a.	Had u liever wel één of beide nieren laten verwijderen?	☐	☐
b.	Is het voornamelijk uw eigen keuze geweest de nier niet te laten verwijderen (ja), of het advies van uw arts (nee)?	☐	☐
c.	Bent u tevreden over die beslissing?	☐	☐

Vul de vraag 2 tot en met 8 in wanneer u wel een nierverwijdering heeft ondergaan.

		erg ontevreden	een beetje ontevreden	geen mening	tevreden	erg tevreden
**2.** a.	Hoe tevreden bent u met het resultaat op korte termijn (tot 1 jaar)?	☐	☐	☐	☐	☐
b.	Hoe tevreden bent u met het resultaat op lange termijn (vanaf 1 jaar)?	☐	☐	☐	☐	☐

3. In hoeverre voldeed de ingreep aan uw verwachtingen?

- ☐ Helemaal niet
- ☐ Een klein beetje
- ☐ Nogal
- ☐ Veel
- ☐ Heel erg veel

4. Kruis aan in hoeverre u het eens bent met de volgende stellingen:

		volkomen oneens	grotendeels oneens	geen mening	grotendeels eens	volkomen eens
a.	Ik had mijn nier liever eerder laten verwijderen	☐	☐	☐	☐	☐
b.	Ik had mijn nier liever later laten verwijderen	☐	☐	☐	☐	☐
c.	Ik had mijn nier liever gelijktijdig met de transplantatie laten verwijderen	☐	☐	☐	☐	☐
d.	Ik had het liefst beide nieren gelijktijdig laten verwijderen	☐	☐	☐	☐	☐
e.	De pijn na de nierverwijdering viel mij tegen	☐	☐	☐	☐	☐
f.	Ik ben gelukkiger sinds mijn nier is verwijderd	☐	☐	☐	☐	☐

5. Hoe lang had u last van pijn na de operatie?

- ☐ Minder dan één week
- ☐ 1-4 weken
- ☐ 4-8 weken (1-2 maanden)
- ☐ 8-16 weken (2-4 maanden)
- ☐ Meer dan 16 weken (4 maanden)

6. **Hoe lang duurde het tot u volledig hersteld was van de ingreep?**

- ☐ Minder dan één week
- ☐ 1-4 weken
- ☐ 4-8 weken (1-2 maanden)
- ☐ 8-16 weken (2-4 maanden)
- ☐ Meer dan 16 weken (4 maanden)

7. **Zou u opnieuw kiezen voor de ingreep, nu u bekend bent met het resultaat?**

- ☐ Ja
- ☐ Nee

a. Zo niet, waar lag dit aan?

- ☐ Het resultaat van de ingreep
- ☐ Tegenvallende vermindering van klachten
- ☐ Tegenvallend cosmetische uitkomst
- ☐ De hoeveelheid pijn na de operatie
- ☐ De duur van de pijn na de operatie
- ☐ Anders, nl ____

8. **Vul deze vraag alleen in als uw nier is verwijderd in de poging bestaande klachten te verminderen. Op een schaal van 1 (geen verandering) tot 10 (klacht compleet verdwenen), in hoeverre zijn uw klachten door de nierverwijdering verminderd? Kruis 0 aan als u geen last had van deze klacht.**

	0	1	2	3	4	5	6	7	8	9	10
a. Drukkend gevoel in de buik	☐	☐	☐	☐	☐	☐	☐	☐	☐	☐	☐
b. Slecht verdragen van eten	☐	☐	☐	☐	☐	☐	☐	☐	☐	☐	☐
c. Weinig eetlust	☐	☐	☐	☐	☐	☐	☐	☐	☐	☐	☐
d. Snel vol gevoel na eten	☐	☐	☐	☐	☐	☐	☐	☐	☐	☐	☐
e. Gewichtsverlies	☐	☐	☐	☐	☐	☐	☐	☐	☐	☐	☐
f. Misselijkheid	☐	☐	☐	☐	☐	☐	☐	☐	☐	☐	☐
g. Overgeven	☐	☐	☐	☐	☐	☐	☐	☐	☐	☐	☐
h. Maagzuurklachten	☐	☐	☐	☐	☐	☐	☐	☐	☐	☐	☐
i. Obstipatie	☐	☐	☐	☐	☐	☐	☐	☐	☐	☐	☐
j. Vermoeidheid	☐	☐	☐	☐	☐	☐	☐	☐	☐	☐	☐
k. Nier-gerelateerde pijn	☐	☐	☐	☐	☐	☐	☐	☐	☐	☐	☐
l. Lever-gerelateerde pijn	☐	☐	☐	☐	☐	☐	☐	☐	☐	☐	☐
m. Bloed in de urine	☐	☐	☐	☐	☐	☐	☐	☐	☐	☐	☐
n. Terugkerende blaasontstekingen	☐	☐	☐	☐	☐	☐	☐	☐	☐	☐	☐
o. Nierstenen	☐	☐	☐	☐	☐	☐	☐	☐	☐	☐	☐
p. Anders, nl ________________	☐	☐	☐	☐	☐	☐	☐	☐	☐	☐	☐

Einde vragenlijst. Hartelijk bedankt voor het invullen.

Nog opmerkingen of toevoegingen?

Vermeldt u deze dan s.v.p. op deze pagina.
